# CRISPR/Cas9-Mediated Knockout of Galactinol Synthase-Encoding Genes Reduces Raffinose Family Oligosaccharide Levels in Soybean Seeds

**DOI:** 10.3389/fpls.2020.612942

**Published:** 2020-12-17

**Authors:** Huy Le, Nhung Hong Nguyen, Dong Thị Ta, Thao Nhu Thi Le, Thao Phuong Bui, Ngoc Thu Le, Cuong Xuan Nguyen, Hardy Rolletschek, Gary Stacey, Minviluz G. Stacey, Ngoc Bich Pham, Phat Tien Do, Ha Hoang Chu

**Affiliations:** ^1^Institute of Biotechnology, Vietnam Academy of Science and Technology, Hanoi, Vietnam; ^2^Division of Plant Sciences, University of Missouri, Columbia, MO, United States; ^3^Leibniz Institute of Plant Genetics and Crop Plant Research, Gatersleben, Germany; ^4^Graduate University of Science, Technology, Vietnam Academy of Science and Technology, Hanoi, Vietnam

**Keywords:** CRISPR/Cas9, *GmGOLS1A*, *GmGOLS1B*, raffinose family oligosaccharides, soybean

## Abstract

Raffinose family oligosaccharides (RFOs) are major soluble carbohydrates in soybean seeds that cannot be digested by human and other monogastric animals. Hence, a major goal is to reduce RFO levels to improve the nutritional quality of soybean. In this study, we utilized a dual gRNAs CRISPR/Cas9 system to induce knockouts in two soybean galactinol synthase (GOLS) genes, *GmGOLS1A* and its homeolog *GmGOLS1B*. Genotyping of T0 plants showed that the construct design was efficient in inducing various deletions in the target sites or sequences spanning the two target sites of both *GmGOLS1A* and *GmGOLS1B* genes. A subset of induced alleles was successfully transferred to progeny and, at the T2 generation, we identified null segregants of single and double mutant genotypes without off-target induced mutations. The seed carbohydrate analysis of double mutant lines showed a reduction in the total RFO content of soybean seed from 64.7 mg/g dry weight to 41.95 mg/g dry weight, a 35.2% decrease. On average, the stachyose content, the most predominant RFO in soybean seeds, decreased by 35.4% in double mutant soybean, while the raffinose content increased by 41.7%. A slight decrease in verbascose content was also observed in mutant lines. Aside from changes in soluble carbohydrate content, some mutant lines also exhibited increased protein and fat contents. Otherwise, no difference in seed weight, seed germination, plant development and morphology was observed in the mutants. Our findings indicate that *GmGOLS1A* and *GmGOLS1B* contribute to the soybean oligosaccharide profile through RFO biosynthesis pathways, and are promising targets for future investigation, as well as crop improvement efforts. Our results also demonstrate the potential in using elite soybean cultivars for transformation and targeted genome editing.

## Introduction

Soybean [*Glycine max* (L.) Merr.] is a major crop grown worldwide with 341 million metric tons produced in 2019 (The American Soybean Association, 2019). In addition to soybean seed oil, which is used extensively in food industry, soybean meal is the number one protein source for animal feed. Soybean seeds can also be used for human consumption. The raffinose family oligosaccharides (RFOs) are a major class of water-soluble carbohydrates present in soybean seeds. Soybean RFOs include raffinose, stachyose and verbascose which, respectively, are mono-, di-, and tri-galactosides of sucrose (Kennedy et al., 1985; Hou et al., 2009), with stachyose being the most prominent RFO in mature seeds. RFOs were proposed to be ubiquitous storage products and factors of desiccation tolerance in soybean seeds, with a potential role in other stress responses (Taji et al., 2002; Wang et al., 2009; Loedolff, 2015; de Souza Vidigal et al., 2016). However, previous studies of soybean seed lines with low stachyose and/or raffinose showed no significant differences in stress tolerance or germination compared to wild-type (Neus et al., 2005; Valentine et al., 2017). RFOs are indigestible by human and other monogastric animals that do not produce α-galactosidase to break the α-1,6-glycosidic bonds in RFOs. These indigestible oligosaccharides go through the stomach to the small intestine and are subsequently fermented by anaerobic microbes residing in the large intestine. The fermentation process produces gases including carbon dioxide and methane, which disturb digestive activity and possibly result in flatulence and diarrhea in monogastric animals (Coon et al., 1990). Therefore, efforts to reduce raffinose and stachyose accumulation in soybean seeds may improve the usefulness of soybean for consumption by humans and monogastric animals, while other seed qualities may remain uncompromised.

The RFO biosynthetic pathway has been well-studied (Peterbauer and Richter, 2001; Panikulangara et al., 2004; Sengupta et al., 2015; Supplementary Figure 1). RFOs are synthesized *de novo* in legume seeds during maturation and desiccation (Kandler and Hopf, 1980) by subsequently transferring the galactosyl group donated from galactinol (1-O-α-d-galactopyranosyl-l-myo-inositol) to sucrose or different RFOs. Galactinol synthase (GOLS) produces galactinol from L-myo-inositol and UDP – galactose and, therefore, is the primary checkpoint of RFO biosynthesis, playing a crucial role in the division of carbon between RFOs and sucrose. In *Arabidopsis thaliana*, *AtGolS1, AtGolS2*, and *AtGolS3* were reported to be differentially induced by abiotic stresses, while overexpression of *AtGolS1, AtGolS2*, or *AtGolS4* correlated with increased galactinol and raffinose accumulation (Taji et al., 2002; Nishizawa et al., 2008). Raffinose is synthesized from sucrose receiving a donated galactosyl, catalyzed by raffinose synthase (RS). Raffinose may receive another galactosyl moiety to become stachyose with the participation of stachyose synthase (STAS) (Saravitz et al., 1987; Peterbauer and Richter, 2001; Nishizawa et al., 2008). Altogether, the expression levels of these enzymes directly contribute to the RFO content in the plant (Taji et al., 2002; Dierking and Bilyeu, 2008). Dierking and Bilyeu (2008) previously localized the causal allele of a low RFO phenotype in soybean to the RS encoding gene *RS2.* A later study found that down regulation of this RS gene by RNA interference silencing significantly reduced raffinose and stachyose with no other changes to plant developmental phenotypes (Valentine et al., 2017). However, applying a RNAi strategy to disrupting RFO biosynthesis has limited economic application. The RNAi silencing effect is transient, likely to be lost over generations, and the presence of transgenic materials in the resulting plant’s genome requires classification as a genetically modified organism (GMO) in most major markets, which may limit commercial appeal.

In this study, we utilized the CRISPR/Cas9 system to induce knockout mutations of two GOLS-encoding homeologs, *GmGOLS1A* and *GmGOLS1B* to study their potential roles in soybean seed RFO metabolism. We generated stable null segregants at the T2 generation and observed interruption of genes coding for GOLS affecting the sugar components of edited soybean seeds. Both single and double mutant knockout seeds showed significant shifts in stachyose, raffinose, verbascose, and sucrose levels. The oligosaccharide phenotypes were achieved without affecting seed germination rate, plant morphology or other key agronomic traits. Hence, our study identifies modification of GOLS expression in soybean as an important target for reducing RFO content in soybean and for improvement of soybean nutritional quality.

## Materials and Methods

### Target Selection and CRISPR/Cas9 Vector Construction

Six putative *GolS* genes were identified from the soybean genome database SoyBase.org by performing BLAST searches against the Arabidopsis GOLS protein sequences. Phylogenetic analysis was conducted to examine phylogenetic relationships among soybean GOLS and other plant species. These data were further combined with protein sequences from NCBI and Plaza databases (Bel et al., 2018). Evolutionary analyses were conducted in MEGA X (Kumar et al., 2018). Protein sequences were aligned by the algorithm MUSCLE, and then compared using the Neighbor-Joining method. Initial trees for the heuristic search were obtained automatically by applying Neighbor-Joining and BioNJ algorithms to a matrix of pairwise distances estimated using a JTT model, with 1,000 bootstrap replicates.

The CRISPR/Cas9-*GmGOLS* transgenic vectors were constructed based on the HBT-pcoCas9 vector, a gift from the Jen Sheen’s laboratory (RRID: Addgene_52254) and the pBlu/gRNA vector, a gift from the Robert Stupar’s laboratory (RRID: Addgene_59188) (Supplementary Figure 4). For each gRNA, DNA oligonucleotides was synthesized by PHUSA Biochemistry (Can Tho, Vietnam) (Supplementary Table 1) and subsequently annealed. The annealed DNAs were individually cloned into *Bbs*I sites of pBlu-gRNA to create pBlu-AtU6-gRNA1 and pBlu-AtU6-gRNA2. These two segments were digested using *Eco*RI, gel purified and subsequently ligated together. Concurrently, the HBT-pcoCas9 vector was digested with *Eco*RI and *Xho*I, and the fragment containing pcoCas9 was assembled into pFGC5941 to create the pFGC-pcoCas9 construct. Finally, the multiplexed gRNA fragments were inserted into *Eco*RI*-*digested pFGC-pcoCas9 to the transgenic vector pFGC-pcoCas9-gRNAs. Sequencing and digestion were performed to validate sequences and orientation of different components in the transgenic vector. The completed construct was mobilized into *Agrobacterium rhizogenes* strain K599 for hairy root transformation and *Agrobacterium tumefaciens* strain AGL1 for soybean stable transformation.

### Soybean Hairy Root Transformation

Soybean hairy root transformation was performed following the *in vitro* method reported by Chen et al. (2018). The constructed CRISPR/Cas9-sgRNA vectors were mobilized into *Agrobacterium rhizogenes* K599 strain via electroporation. Cotyledons from 3-days old seedlings of Maverick and DT26 cultivars were used as explants for bacterial infection. Soybean hairy roots, formed at 10–15 days after co-cultivation, were transferred to selection medium (MS medium with 3 mg/l Glufosinate). Ten herbicide-resistant hairy roots, along with wild-type roots induced by K599 without the constructed vector, were chosen for genomic DNA extraction using the CTAB method (Dellaporta et al., 1983) and used for PCR amplification of target regions. The PCR products from each root were mixed with wild-type amplicons, subjected to DNA denaturation followed by renaturation, and analyzed using polyacrylamide gel electrophoresis (PAGE). Amplicon of regions that contained polymorphic alleles to wild-type, following the treatment above would result in heteroduplexes which travel more slowly during gel electrophoresis, manifesting as distinct bands above that of the wild-type, homoduplex DNA band (Zhu et al., 2014).

### Stable Soybean Transformation and Transgene Confirmation

Two soybean cultivars, DT26 and Maverick, were used for cotyledon node *Agrobacterium-*mediated transformation following an established procedure (Margie et al., 2004). Regenerated plants on selection medium were screened by herbicide leaf-painting (200 mg/l Glufosinate) onto trifoliate leaves. Leaf painting was done three times for each regenerated plant. Leaves of herbicide-resistant plants were collected for genomic DNA extraction following the original CTAB method (Dellaporta et al., 1983). PCR reactions with primer pairs specific for pco*Cas9* gene and transgene’s 35SPPDK promoter region (Supplementary Table 1) were performed to validate transgene presence in edited plants.

### Plant Materials, Plant Growth Conditions, and Morphological Characterization

This study was conducted using the soybean cultivar Maverick and Vietnamese elite cultivar DT26. Soybean seeds were imbibed on moist paper placed in a petri dish for 48 h at 28°C. Germinated seeds were subsequently sown in plastic pots (25 cm tall, 20 cm diameter) and planted under greenhouse conditions at 28–35°C, with a 16:8 h day and night photoperiod. Each pot contained TRiBAT compost mixture (Green Saigon Biotech, Vietnam). Soybean plants were fertilized two times with NPK (15:5:20) when 40 days-old and with NPK (16:16:16) at 65 day-old. Seeds were harvested and stored in a long-term seed storage compartment (40% humidity, 4°C) for further experiments. Plant growth parameters, including plant height (cm), branch number, and internode number were recorded when each soybean plant had reached maturity (R8 stage). Leaf length (cm) and leaf width were measured using different leaflets at the R2 stage of soybean development.

### Induced Mutant Identification and Characterization

The spanning DNA regions containing the two targets on *GmGOLS* genes were amplified by flanking gene-specific primer pairs (Supplementary Table 1). The amplicons were analyzed for induced mutations using agarose gel electrophoresis for large indels (Do et al., 2019) and PAGE for small indels (Zhu et al., 2014). PCR amplicons were subsequently purified and ligated to the pJET1.2 cloning vector (Thermo Fisher Scientific, United States) for sequencing by ABI PRISM^®^ 3,100 Avant Genetic Analyzer system with Applied Biosystems Big Dye Terminator cycle sequencing kit (Applied Biosystems, United States) at the Institute of Biotechnology, Hanoi, Vietnam. The sequences were compared to wild-type sequences using the FinchTV chromatogram viewer program (Geospiza) and MEGA X (Kumar et al., 2018). For analysis of potential off-targets indicated by the online program CCTop (Stemmer et al., 2015), genomic DNA from those T2 mutant lines showing an altered carbohydrate profile and their T2 siblings’ were bulked together, and the flanking regions of putative off-target loci were PCR amplified and sequenced by site-specific primers (Supplementary Table 1).

### Determination of Seed Composition

Measurements of soluble carbohydrates in T3 seeds from greenhouse-grown T2 lines were performed by ion chromatography using pulsed amperometric detection (ICS-3000, Thermo Fisher Scientific, United States). Mature seed samples were grounded and extracted using methanol (80% v/v) as described by Weber et al., 2000. Separation of the sample extract was carried out on a PA1 column (2 mm × 250 mm) with a guard column (PA1 2 mm × 50 mm) at 40°C by applying an isocratic run with 100 mM NaOH at a constant flow rate of 0.35 mL/min. Compound identities were verified by matching retention time to authenticated standards. External calibration was applied using authenticated standards.

The seed contents of fat, starch, protein and moisture were measured in ground mature seed samples using near-infrared spectroscope (MPA; Bruker, United States), calibrated according to the supplier’s protocol.

### Seed Germination Test

Seed germination test was performed according to the methods outlined by Blöchl et al. (2007) for the water treatment. Seeds of T2 single *gmgols1A* mutant (DT1.1-7-2, DT1.1-14-1, DT1.1-14-3) and *gmgols1A gmgols1B* double mutants (DT1.1-5-3, DT1.1-13-1, DT1.1-14-10) along with wild-type DT26 control seeds were used for the experiment. The experiment was conducted with three replicates for each genotype with 50 seeds per replicate. Germination rate was recorded every 24 h until 120 h, where 0 h is the time of imbibition.

### Statistical Analysis

Sample means between genotypes were compared using Student *t*-test or one-way ANOVA followed by a *post hoc* Tukey’s multiple range test. Statistical significance was considered at *p* = 0.05. For HPLC seed compositional analysis, each induced mutant genotype was represented by seeds from two lines. Quadruplicates samples were measured for each line. All statistical analyses were performed using SPSS software version 24.1.

## Results

### Target Selection and Mutagenesis Confirmation in Soybean Hairy Roots

As shown in Supplementary Figure 2, the six *GmGOLS* genes comprise three homeologous pairs, one pair clustered with *AtGOLS1* and two pairs clustered with *AtGOLS2/3.* The expression pattern of the various *GmGOLSs* genes was previously determined in different tissues and seed developmental stages^1^ (Supplementary Figure 3; Joshi et al., 2014). We found that *Glyma.03G222000 (GmGOLS1A)* expression increases sharply as the soybean seed matures and shows the highest expression level in seeds among all *GmGOLS* genes (Supplementary Figure 3). The *GmGOLS1A* homeolog, *Glyma.19G219100 (GmGOLS1B)* gene, was initially expressed at a similar level to the *GmGOLS1A* gene at early stages of seed development, i.e., up to seed stage S6, but expression decreased during later stages. These expression data, therefore, indicate that *GmGOLS1A* is likely the major GOLS enzyme involved in RFO biosynthesis during soybean seed development. Hence in this study, we mutagenized *GmGOLS1A* and its homeolog, *GmGOLS1B*, using a CRISPR/Cas9 approach to examine the function of GmGOLS in soybean seed RFO biosynthesis.

**FIGURE 1:**
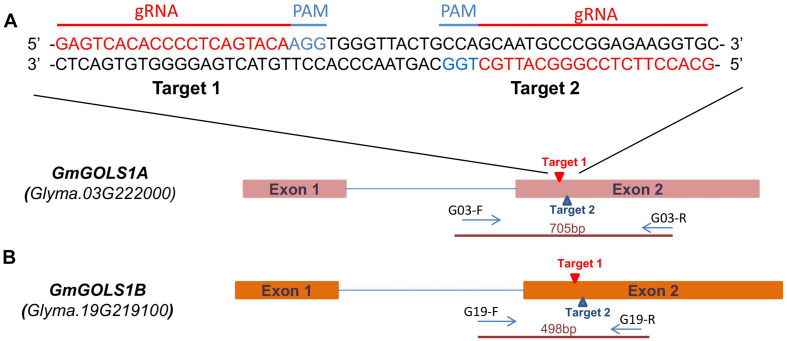
*GmGOLS* gene structure and target sequence locations. **(A)** sgRNA and PAM sequences on targeted genes. **(B)** *GmGOLS1A, GmGOLS1B* gene structure, targeted sites on *GmGOLS1A, GmGOLS1B*, and location of primer pairs used in genotyping for induced mutations.

Based on the Williams 82 reference genome, two single guide RNA (sgRNAs) were selected from potential target sites on *GmGOLS1A* and *GmGOLS1B*. These two target sequences are identical and located within exon 2 of both *GmGOLS* genes (Figures 1A,B). We selected a Vietnamese elite soybean (DT26) and Maverick cultivar as explants for targeted mutagenesis. Preliminary sequencing found that the targeted sequences in *GmGOLS1A* and *GmGOLS1B* genes encoded in Maverick and DT26 were identical to those in the reference genome William 82 (Schmutz et al., 2010).

To validate the efficacy of the CRISPR/Cas9 vector construct in inducing targeted deletions, we mobilized this plasmid construct into *Agrobacterium rhizogenes* strain K599 for hairy root transformation following the method outlined in a previous report (Chen et al., 2018). The denaturation-renaturation analysis on PAGE of PCR amplicons from 12 tested hairy roots showed mobility shifted bands indicating induced mutations for both target genes in 11 samples; one sample showed shifted bands only for the region on *GmGOLS1B* but not on *GmGOLS1A* (Supplementary Figure 5). Sequencing data (not shown) confirmed that various indels occurred at the targeted sites demonstrating the high efficacy of our CRISPR/Cas9 construct for stable soybean transformation.

### Induced Mutations of *GOLS* Genes in T0 Transgenic Soybean

**FIGURE 2:**
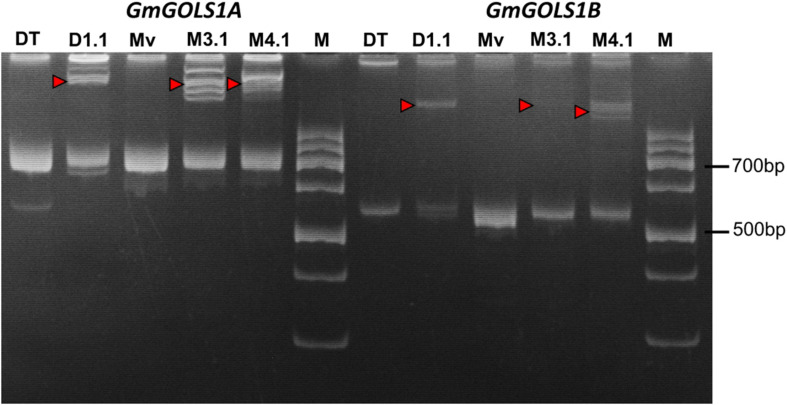
Induced mutation analysis using denaturation and renaturation PCR – polyacrylamide gel electrophoresis. Red triangles denote heteroduplex bands indicating heterozygosity with wild-type sequence. (Mv) wild type plants of Maverick cultivar; (DT) Wild type plants of DT26 cultivar; (M3.1, M4.1) mutant lines of Maverick cultivar; (D1.1) mutant line of DT26 cultivar; (M) 100 bp DNA ladder.

Following the *Agrobacterium* mediated transformation procedure using soybean cotyledonary nodes, we generated three primary (T0) transgenic lines: two from the Maverick cultivar (M3.1, M4.1) and one from the DT26 cultivar (DT1.1). Successful transformants were identified initially by herbicide leaf painting (200 mg/l Glufosinate), then confirmed by PCR screening for the presence of the T-DNA sequence using specific primers for the *pcoCas9* gene and a 35SPPDK – pFGC spanning region within the transgene (Supplementary Table 1). We subsequently amplified the DNA sequences spanning the targeted regions in *GmGOLS1A* and *GmGOLS1B* from transgenic plant DNA, and performed PAGE of the denatured and renatured PCR products (Figure 2). Mobility-shifted bands, heteroduplexes formed from the heterogenous amplicons, occurred in all three tested T0 samples for both genes but not in their respective wild-type control, indicating the high efficacy of soybean genome editing utilizing our designed CRISPR/Cas9 constructs.

**FIGURE 3:**
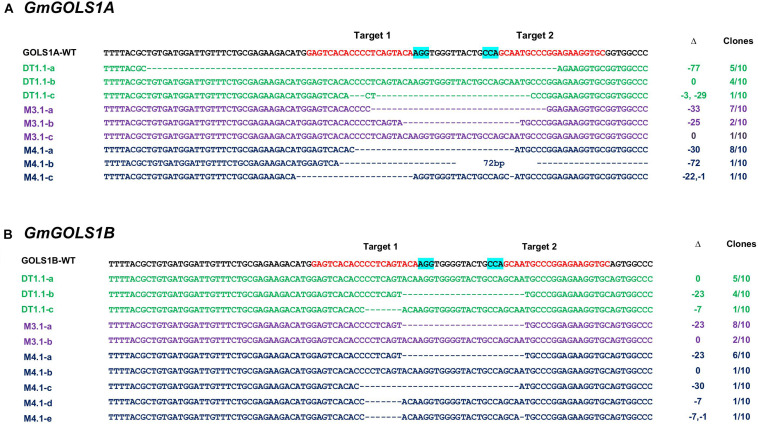
Genotyping of targeted regions in *GmGOLS1A* **(A)** and *GmGOLS1B* **(B)** genes in T0 plants. Targeted sequences (sgRNAs) are indicated by red color; the PAM sequences are highlighted in blue. (D1.1) mutant line of DT26 cultivar; (M3.1, M4.1) mutant lines of Maverick cultivar; a/b/c/d/e indicates different alleles for each T0 line; Δ indicates targeted sequence changes: 0 for no change, −for deletion, +for insertion. Clones indicate the number of clones sequenced for each respective allele, out of the total sequenced clones.

To validate and characterize the CRISPR/Cas9-induced mutations in the target genes, we sequenced PCR amplicons of DNA sequences spanning the targeted *GmGOLS* regions in T0 plants. Consistent with the PAGE analysis results, the sequencing data showed various induced mutations in the two *GmGOLS* genes (Figure 3). The deletion size varied from Δ-7 to Δ-77 nucleotides. The DT1.1 event contained chimeric indels for both *GmGOLS1A* and *GmGOLS1B* genes. Two deleted indels (Δ-77 and Δ-32 nucleotides) as well as wild-type sequences in the *GmGOLS1A* gene were identified from the DT1.1 event. In addition, we also found wild-type allele and two induced mutant alleles (Δ-7 and Δ-23 nucleotides) in the *GmGOLS1B* gene in this T0 transgenic event. The expected deletions in the *GmGOLS1A* and *GmGOLS1B* genes were also observed in M3.1 and M4.1 transgenic lines from the Maverick cultivar. Interestingly, all three transgenic lines contained an identical Δ-23 mutation between the two expected Cas9 cut sites within the *GmGOLS1B* gene. In addition, most of the detected indels (8/9) were found between the two target sites, indicating the precision of the designed CRISPR/Cas9 construct for soybean genome editing.

Polyacrylamide gel electrophoresis and sequencing analysis indicated that all generated T0 transgenic soybeans (3/3) from both DT26 and Maverick cultivars carried CRISPR/Cas9 induced mutations in *GmGOLS1A* and *GmGOLS1B* genes. Aside from M3.1’s *GmGOLS1B* locus, all other regions contained two or more mutant alleles, requiring further screening of T1 generation progeny to identify stably inherited mutations.

### Inheritance and Segregation of Induced Mutants

**FIGURE 4:**
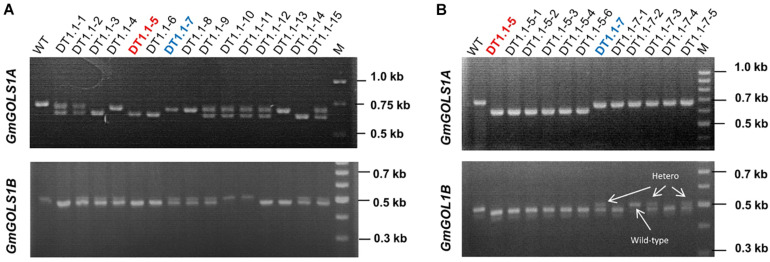
Analysis of targeted mutant segregation of T1 and T2 plants from the D1.1 line. **(A)** and **(B)** PCR – gel electrophoresis of targeted regions in *GmGOLS1A* and *GmGOLS1B* genes at T1 and T2 generation. WT, wild type plants of DT26 cultivar; DT1.1-1 to DT1.1-15, T1 plants from D1.1 mutant line; (M) 1kb DNA ladder on the T1 gels and 100bp DNA ladder on the T2 gels; DT1.1-5-1 to DT1.1-5-6, T2 plants from DT1.1-5; (DT1.1-7-1 to DT1.1-7-5) T2 plants from DT1.1-7.

We planted T0 lines under greenhouse conditions and collected seeds to assess the inheritance of mutant *GmGOLS* alleles at T1 generation. We successfully identified the segregation of big deletions (Δ-23 and Δ-77 bp) of *GmGOLS1A* and *GmGOLS1B* genes in progenies of the DT1.1 transgenic line using agarose gel electrophoresis (Figure 4). For *GmGOLS1A*, seven of fifteen tested T1 plants showed double bands; however, they were all lower than the amplified band of wild-type plant (Figure 4A). In addition, four T1 plants carried only the upper band and the rest displayed only the lower band, suggesting homozygous genotypes. For the *GmGOLS1B* gene, eight T1 plants had double bands, while, five plants carried only the lower band and two plants showed the upper band (Figure 4B). Importantly, two T1 plants (DT1.1-5 and DT1.1-6) displayed the lower bands for both *GmGOLS1A* and *GmGOLS1B* genes, indicating that these lines carried large deletions of both *GmGOLS* genes.

**FIGURE 5:**
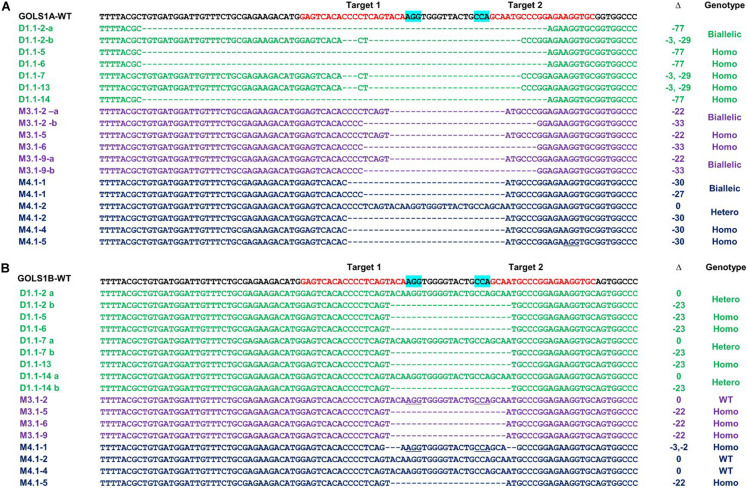
Inheritance of targeted mutations in T1 *GmGOLS1A* **(A)** and *GmGOLS1B* **(B)** plants from DT1.1, M3.1, and M4.1 lines. a/b indicates different alleles in heterozygotes. Δ indicates the targeted sequence changes: 0 for no change, −for deletion, +for insertion. The genotypes of T1 mutant lines were classified as biallelic for two different mutant alleles, hetero for heterozygotic with one wild type allele and one mutant allele, homo for homozygotes with two identical mutant alleles.

Sequencing data of the selected T1 progenies DT1.1-2, DT1.1-5, DT1.1-6, DT1.1-7 DT1.1-13, DT1.1-14 derived from the DT1.1 T0 event showed that DT1.1-5, DT1.1-6, and DT1.1-13 were double mutants for both *GmGOLS* genes, while others (DT1.1-2, DT1.1-7, and DT1.1-14) were biallelic/homozygous for *GmGOLS1A* and heterozygous for *GmGOLS1B* (Figure 5). The targeted sequences of *GmGOLS1A* in T1 offspring were consistent with the agarose gel electrophoresis analysis (Figure 4). DT1.1-5, DT1.1-6, and DT1.1-14 plants carried the Δ-77 bp mutant allele, while DT1.1-7 and DT1.1-13 plants inherited the Δ-32 bp allele. In addition, the DT1.1-2 line contained both Δ-32 bp and Δ-77 bp deletions (biallelic). Sequencing of the *GmGOLS1B* gene showed that T1 progeny carried the wild-type allele and a Δ-23 bp allele; DT1.1-5, DT1.1-6, DT1.1-13 were homozygous mutants while DT1.1-2, DT1.1-7, and DT1.1-14 showed heterozygous mutations. Importantly, no wild-type allele of *GmGOLS1A* gene was observed in all tested T1 progeny, despite its apparent presence at the T0 generation (DT1.1) (Figures 3, 5). This outcome regretfully indicated that we were not able to obtain *gmgols1B* single mutant plants and subsequent phenotypic characterizations were done on single mutant *gmgols1A* and double mutant *gmgols1A gmgols1B* plants.

The stable inheritance of all induced mutant alleles of both *GmGOLS1A* and *GmGOLS1B* genes in the DT1.1 line was confirmed at the T2 generation (Figure 4 and Supplementary Figure 6). The tested T2 plants were homozygous for the Δ-32 bp or Δ-77 bp mutant alleles of *GmGOLS1A*, which were found in T1 mutant lines (DT1.1-5; DT1.1-7; DT1.1-13). The induced mutation of *GmGOLS1B* continued to segregate among T2 plants from the heterozygous T1 lines DT1.1-4 and DT1.1-7. Of these, DT1.1-4-5 and DT1.1-7-1 had the homozygous Δ-23 bp *GmGOLS1B* genotype, whereas DT1.1-4-3 and DT1.1-7-2 was wild-type for the same gene.

The inheritance of CRISPR/Cas9-induced mutations in M3.1 and M4.1 offspring, generated in cultivar Maverick, was also investigated by gel electrophoresis and sequencing (Figures 3, 5). At the T0 generation (Figure 3), we found that both M3.1 and M4.1 lines carried chimeric mutations of both *GmGOLS1A* and *GmGOLS1B* genes. However, most of the mutations were not passed to progeny. For *GmGOLS1A*, only certain alleles that occurred at T0 (M3.1 Δ-33 bp and M4.1 Δ-30 bp) were inherited, while new alleles were also observed at the T1 generation. In addition, we detected all new mutant alleles of the *GmGOLS1B* gene in T1 progeny which were not observed in T0 plants. Importantly, the newly induced mutant alleles occurred as homozygous and biallelic forms in T1 progeny, which were subsequently stably inherited in the T2 generation (data not shown).

### Composition of *GmGOLS* Mutant Soybean Seeds

As mentioned above, GOLS catalyzes the first step in the RFO biosynthetic pathway (Nishizawa et al., 2008). Therefore, we hypothesized that knock-out mutations in the *GmGOLS* genes, specifically in *GmGOLS1A* that encodes the major GOLS expressed in developing soybean seeds, would affect the final sugar composition in mature seeds.

**FIGURE 6:**
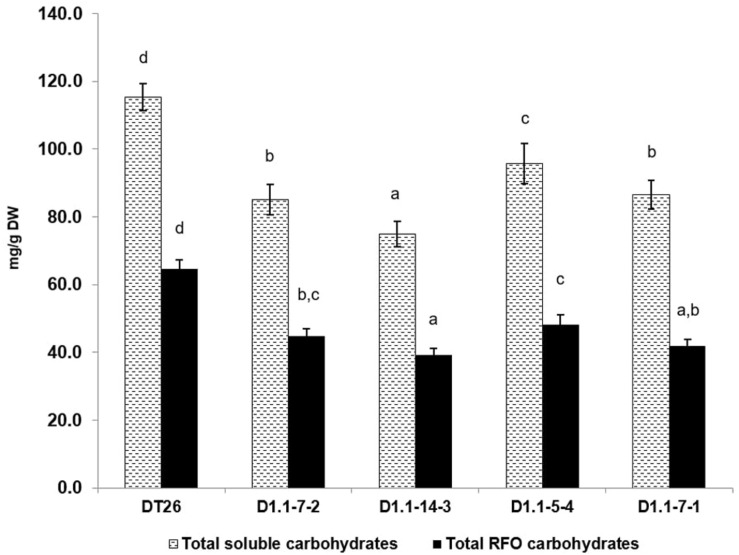
Total soluble carbohydrates and total RFOs as measured by HPLC. Wild-type seeds are of DT26 cultivar; D1.1-7-2, D1.1-14-3 are seeds from T2 *gmgols1A* single mutant; D1.1-5-4, D1.1-7-1 are seeds from T2 *gmgols1A gmgols1B* double mutants. Statistical analysis was done using one-way ANOVA followed by a *post hoc* Tukey’s multiple range test. Measurements that were not significantly different from one another (*p* > 0.05) share the same letter label (a,b,c). Mean values ± SD for *n* = 4 are shown.

**FIGURE 7:**
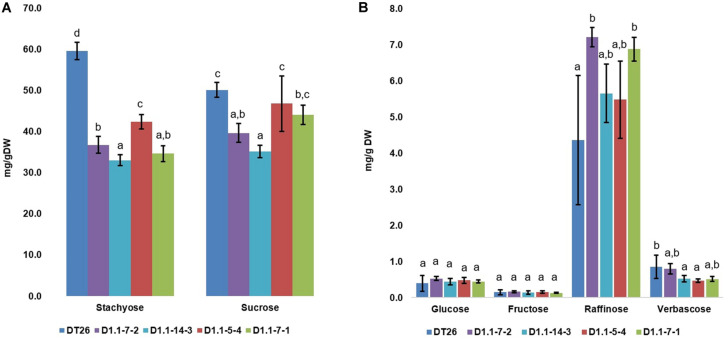
Carbohydrate composition in soybean seeds, with the major carbohydrates stachyose and sucrose in **(A)** and less abundant carbohydrates in **(B)**. Wild-type seeds are of DT26 cultivar; D1.1-7-2, D1.1-14-3 are seeds from respectively named T2 *gmgols1A* single mutant; D1.1-5-4, D1.1-7-1 are seeds from T2 *gmgols1A gmgols1B* double mutants. Statistical analysis was done using one-way ANOVA followed by a *post hoc* Tukey’s multiple range test. Measurements that were not significantly different from one another (*p* > 0.05) share the same letter label (a,b,c). Mean values ± *SD* for *n* = 4 are shown.

We further analyzed the seed components of selected mutant lines using chromatography (Figures 6, 7). Compared to wild-type seeds, tested mutant seeds all had reduced RFO sugar and total soluble carbohydrate content. The reduction of total soluble carbohydrates was mainly driven by decreases in stachyose and sucrose, which together account for 90% of total quantified soluble carbohydrates in wild-type seeds. Compared to stachyose in wild-type seeds (59.5 mg/g DW), mutant lines showed a reduction of 41.4% to 34.86 mg/g DW in single mutants (*p* < 0.001), and 35.4% to 38.46 mg/g DW in double mutants (*p* < 0.001). However, no significant difference in stachyose was observed between single and double mutant lines (*p* = 0.0679). Sucrose content in mutant lines also decreased by 25.4% to 37.4 mg/g DW in single mutants (*p* < 0.001) but did not change significantly in double mutants (9.4% decrease to 45.4 mg/g DW, *p* = 0.1) compared to seeds from wild-type plants (50.1 mg/g DW). The content of other RFOs also differed significantly from wild-type. All tested mutant lines exhibited increased raffinose accumulation, which was on average 47.44% higher in single mutants (*p* = 0.0254) and 41.7% in double mutants (*p* = 0.0472). However, the raffinose content of single and double mutants was not statistically different (*p* = 0.24). Moreover, verbascose content decreased by 22.6% (single mutant seeds) and 42.1% (double mutant seeds) compared to wild-type plants. Total RFOs content, measured as the sum of raffinose, stachyose, and verbascose, was 64.7 mg/g DW in wild-type, but reduced by 30.2% to 45.13 mg/g DW in single mutants, and by 34.1% to 41.95 mg/g DW in double mutants. No significant change in glucose or fructose content was found between wild-type and mutant seeds (*p* > 0.05).

Interestingly, when individual carbohydrate levels were calculated as a proportion of total soluble carbohydrates (Supplementary Figure 7), it was apparent that the relative proportion of sucrose actually increased in both the single and the double mutants as compared to the wild-type. In addition, the stachyose proportions in double and single mutants were lower as compared to wild-type plants.

We also performed quantitative analysis of other seed components including moisture, protein, fat, and starch (Supplementary Figure 8 and Supplementary Table 2). No significant difference in seed starch composition between tested mutant seeds and wild-type seeds was observed. However, seeds of *gmgols1A* single mutant lines had increased protein (40.06% to wild-type’s 38.30%, *p* = 0.00254) and fat content (21.9% to wild-type 19.8%, *p* = 0.0201), while no change was observed in *gmgols1Agmgols1B* double mutant lines (*p* = 0.581 and 0.406 for protein and fat, respectively). This result indicated that the function of *GmGOLS1A* and *GmGOLS1B* may be not restricted to RFO biosynthesis, and further investigations need to be performed to study apparent pleiotropic effects on overall biosynthetic activities determining major biomass components.

### Mutant Soybean Growth and Morphology

**FIGURE 8:**
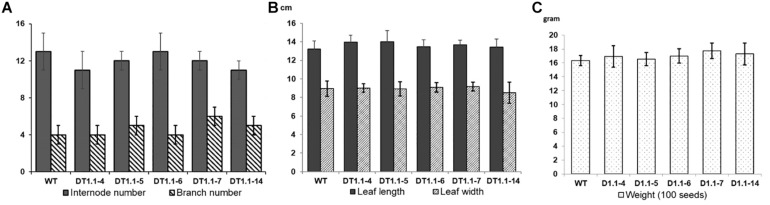
Graphs of growth parameters and seed weight of T2 *GmGOLS* mutant soybean plants. **(A)** Internode number and branch number of 90-day-old plants, **(B)** size of leaf (including leaf length and leaf width) of 90-day-old plants, **(C)** The 100 seed weight of tested lines. Error bars indicate standard deviation. On **(A)**, *X* axis indicates the number of internode or branch, respectively; On **(B)**, *X* axis indicates measurement in centimeter (cm); On **(C)**, *X* axis indicates seed weight in gram. Label indicates name of parent (T1) plants, with WT being wild type DT26.

Selected homozygous T2 mutant soybeans (progeny of DT1.1-4, DT1.1-5, DT1.1-6, DT1.1-7, DT1.1-14) and wild-type DT26 plants were cultivated under greenhouse conditions for morphological analysis (Figure 8 and Supplementary Figure 9). We found no significant differences in plant morphology between *gmgols* mutant lines and wild-type plants. The same internode number (13 ± 1) was observed in mutant and wild-type plants. The branch number varied slightly between wild-type plants (5 ± 1) and mutant lines (4 ± 1), but this was not statistically significant (Figure 8A). Furthermore, the average leaf length and width of mutant lines were also comparable to those of wild-type plants, again no statistically significant differences were observed (Figure 8B). Under greenhouse condition, the tested *gmgols* mutant soybeans showed no change in seed weight as compared to wild-type plants (Figure 8C). Altogether, these data indicate that knock-out mutations in *GmGOLS1A and GmGOLS1B* had no deleterious effects on soybean growth under greenhouse conditions.

### Germination of Knockout Mutants

**FIGURE 9:**
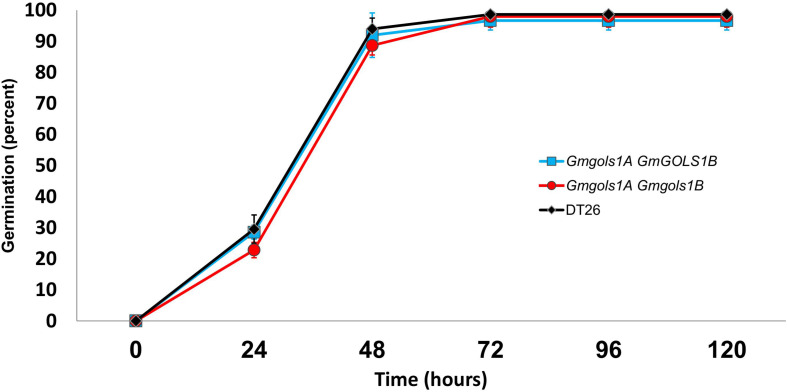
Germination of wild type (DT26) and selected *GmGOLS* mutant seeds. Data points are averages of three biological replicates, *n* = 50. Error bars indicate standard deviations.

To assess seed vigor, researchers often utilize a seed germination test under normal germination conditions, or with additional treatments, such as high heat and humidity in the case of the accelerated aging tests (TeKrony, 2005). Previous reports also utilized an imbibition method with 1-deoxygalactonjirimycin (DGJ), a specific α-galactosidase inhibitor, to study the role of RFOs in seed germination (Blöchl et al., 2007). In the current study, we performed a germination test of water-imbibed T3 seeds from DT1.1 selected single *gmgols1B* and double *gmgols1A/gmgols1B* mutant plants (Figure 9). We observed a marginally lower rate of germination for mutant seeds at 24 h (29 ± 2% for single mutants, 23 ± 2% for double mutants, 30 ± 5% for wild-type) and 48 h (92 ± 7% for single mutants, 89 ± 3% for double mutants, 94 ± 3% for wild-type). However, these variances were not statistically significant. At 96 h, the germination rate of all groups was stable at over 95%. Altogether, under the tested conditions, we found no significant difference in germination between the *gmgols* mutant and wild-type seeds.

### Identification of Transgene-Free Homozygous Mutants

We determined the inheritance of transgenes from T0 generation to T2 by screening for transgene-conferred herbicide resistance and PCR analysis of genomic DNA (Supplementary Figure 10 and Supplementary Table 3). At the T1 generation, all 29 tested T1 plants of DT1.1 line were resistant to herbicide, and the transgene presence was subsequently confirmed by PCR analysis with pco*Cas9* and 35SPPDK-specific primers. On the other hand, transgene-free genotypes were identified in several T1 plants derived from M3.1 and M4.1 transgenic lines. We subsequently screened T2 progeny of DT1.1 line and identified transgene-free mutant plants. Of which, DT1.1-7-1 was characterized as a double *gmgols* mutant null segregant, and DT1.1-7-2 carried a single null mutation of the *GmGOLS1A* gene. Importantly, all T3 plants produced from these two T2 transgene-free mutant lines were susceptible to the herbicide leaf-painting test. PCR analysis also demonstrated the absence of the *bar* and *Cas9* genes in these T3 plants.

### Off-Target Analysis

Off-target activity has been reported in several studies using the CRISPR/Cas9 system (Sun et al., 2015). During the process of target selection using the online program CCTop (Stemmer et al., 2015), we identified two likely off-target sites locating in the *Glyma.07G220600* and *Glyma.09G169400* genes, respectively. Both the genome sequence of the reference William 82, as well as the genome of tested cultivars (DT26 and Maverick), contain these potential off-target sites with identical PAM sequences and 3–4 nucleotide mismatches to either of the targets used (Supplementary Table 4). We performed off-target analysis on T2 and T3 mutant lines by sequencing the off-target flanking regions (Supplementary Figure 11). We detected no editing activity at these off-target sites, indicating the high gene specificity of CRISPR/Cas9 editing in soybean.

## Discussion

### Selection of Target Sites for Dual Induced Deletions

Simultaneous mutagenesis of two or more homeologs in soybean by the same guide RNA has been successfully achieved since early attempts to utilize CRISPR/Cas9 in this paleopolyploid species (Jacobs et al., 2015; Kanazashi et al., 2018). We also pursued the double gRNAs cleavage strategy, as previously described in rice, tomato and soybean, for its ability to generate dual induced deletions that increases the likelihood of obtaining functional knockouts that can be easily identified with gel electrophoresis (Brooks et al., 2014; Zhou et al., 2014; Do et al., 2019). With these criteria in mind, we narrowed the selection of target sites that were specific to *GmGOLS1A* and *GmGOLS1B*. The conserved and identical target sites were limited to the exon 2 region in both selected genes. These targeted sites were expected to produce deletions of 23–25 base pairs, relatively short compared to previous efforts employing the dual gRNAs to cleave target sites at long distance (Xie et al., 2015; Do et al., 2019). As a result, the short distance between cleavage sites improved the rate of simultaneous cleavage by the Cas9 protein. Specifically, the rate of large deletions spanning two cleavage sites was 80% for *GmGOLS1A* gene and 63.3% for *GmGOLS1B* gene from three T0 plants (Figure 3). In contrast, small indels of fewer than five nucleotides at each target site occurred at a very low rate (3.3% at *GmGOLS1A* and 10% at *GmGOLS1B*). Consistent with previous reports (Xie et al., 2015; Minkenberg et al., 2017), our study further indicated the importance of target distance selection for simultaneously induced cleavages using the CRISPR/Cas9 system.

### Late CRISPR/Cas9 Editing Activity in T0 – T1

For the DT1.1 mutant line, all alleles found in the T1 generation were observed at the T0 (Figures 3, 5) generation. In addition, these mutations were stably inherited in the T2 generation. However, T1 plants from other transgenic lines (M3.1, M4.1) carried several mutant alleles not found at the T0 generation (Figures 3, 5). As reported in multiple studies (Curtin et al., 2018; Kanazashi et al., 2018), CRISPR/Cas9 mutagenesis observed in the soybean T0 generation does not always correspond to mutations inherited at the T1 generation. The inconsistency in our results could have been due to CRISPR activity occurring independently late in the development of T0 plants, which would result in chimeric mutations in tissues collected for T0 plant analysis. Unidentified mutations in other tissues could have been inherited in T1 plants. This phenomenon was previously well-documented by Kanazashi et al. (2018), in which DNA analysis of each individual node was performed and their observed alleles matched with that of the seeds from the respective nodes. Future genome editing studies using soybean and other recalcitrant plant species may likewise benefit from this approach.

### Seed Germination

Raffinose family oligosaccharides were proposed to have many functions in seed storage and germination, such as providing energy or protection from environmental stress (Peterbauer and Richter, 2001; Loedolff, 2015; Sengupta et al., 2015). A previous study identified a positive correlation between total seed RFO content and seed vigor in *Arabidopsis thaliana* and maize (*Zea mays*), whereas sucrose content was negatively correlated to seed health (Li T. et al., 2017). In another study of pea seeds, RFO breakdown was found to be necessary for germination (Blöchl et al., 2007). However, using different methods with water or DGJ, Dierking and Bilyeu (2009) considered RFOs as a non-essential energy source during seed germination of soybean. In our current study, a water-imbibed germination test was performed. Mutant seeds with altered sucrose and RFO contents showed no significant differences in seed vigor compared to wild-type seeds under the conditions we tested (Figure 9). This result indicates that the changes in seed sugar components of soybean *gmgols* mutant lines had no effects on seed health and germination, consistent with previous results using RNAi RFO soybean lines.

### Phenotypes of *gmgols* Induced Mutants Demonstrate Modified Carbohydrate Profiles

Previous reports indicated that natural mutations or RNAi-mediated silencing of *RS* genes resulted in low RFOs phenotypes in soybeans (Kerr and Sebastian, 2000; Dierking and Bilyeu, 2008; Valentine et al., 2017). In this study, the two *GmGOLS* genes coding for GOLS, an enzyme involved in the immediate step before raffinose biosynthesis in the RFOs metabolism pathway (Peterbauer et al., 2001; Panikulangara et al., 2004; Sengupta et al., 2015), were subjected to induced mutation using the CRISPR/Cas9 system. *gmgols* mutant lines showed significant decreases in the total RFO content compared to wild-type (Figure 6). However, the changes were different for each RFO component (Figure 7). Raffinose levels increased by up to 65%, while stachyose and verbascose both decreased by as much as 45%. Since stachyose is the major RFO in soybean seeds, its reduction in edited lines drove the total RFO amount in seeds down by 30.2% in single mutants and 35.2% in double mutants. Unexpectedly, sucrose levels also decreased by a significant amount in *gmgols1A gmgols1B* single mutants, but not in *gmgols1A gmgols1B* double mutants (Figure 7A).

The soybean cultivar PI 200508 was identified as a low stachyose and raffinose introduction line (Kerr and Sebastian, 2000). Using this line, Dierking and Bilyeu (2008) identified *RS2* as the gene bearing the causal allele for decreased RFO content in PI 200508. Valentine et al. (2017) later demonstrated that knockdown of *RS2* resulted in a three-fold reduction of stachyose and five-fold reduction of raffinose as well as a 50% increase of sucrose in seeds from greenhouse grown plants. In this study, we observed that a *GOLS* mutation differentially affected sucrose, raffinose, and stachyose accumulation. Most importantly, the increase of raffinose, and the concurrent decrease of stachyose (its downstream product) suggests the existence of an unknown regulatory bias for RFOs in the soybean seed that involves GOLS function. Future investigations should further detail the carbohydrate profile of the generated transgenics under different growth conditions, quantify more relevant substrates such as myo-inositol and galactinol, and possibly combine known causative genotypes (e.g., RS2 mutant alleles).

As we compared RFO profiles of *gmgols1A* single mutants and *gmgols1A gmgols1B* double mutants, we observed several phenotypic differences that may be attributed to *gmgols1B* loss-of-function (Figures 6, 7 and Supplementary Figure 7 and Supplementary Table 2). As measured by dry weight, sucrose levels in *gmgols1A* single mutant seeds exhibited a significant reduction compared to wild-type seeds, while that in *gmgols1A gmgols1B* double mutant seeds was similar to wild-type seeds. Intriguingly, *gmgols1A* single mutant seeds also had increased protein and fat content, while double mutant seeds exhibited no difference. Otherwise, no difference in RFO content was found between *gmgols1A* single mutant and *gmgols1A gmgols1B* double mutant seeds. Altogether, our data suggests that GmGOLS1A/1B may impact more than strictly RFO metabolism.

As RFOs are still generated in substantial amounts in the mutant seeds, we must assume that there is significant residual activity of the GOLS enzyme. This might be attributed to the other *GOLS*-encoding genes with high expression in the seed that were not characterized in this study, in particular *Glyma.19G227800* and *Glyma.10G145300*. Additional knock-out of these genes (in our double mutant background) is expected to dramatically impact overall RFO biosynthesis.

### Potential With Elite Cultivar

Soybean is a recalcitrant crop for transformation and genetic engineering. Previous reports demonstrated transformation successes with only certain soybean cultivars including Williams 82, Jack, Thorne, and Maverick (Yamada et al., 2012; Xie et al., 2015; Li S. et al., 2017). Here, we present results on soybean transformation and genome editing using the elite soybean cultivar DT26. Transgene-free and homozygous mutant lines were observed at the T2 generation, indicating the potential of *Agrobacterium*-mediated method and the CRISPR/Cas9 system for improvement of other soybean elite cultivars and other important crops. Depending on the regulations of each country, this provides the potential to create non-GMO, edited elite soybean lines that can be directly utilized for crop protection after the appropriate regulatory approval.

## Conclusion

We successfully induced knock-out mutations in *GmGOLS1A* and *GmGOLS1B* genes using a dual gRNA CRISPR/Cas9 system. At the T2 generation, we acquired null segregants which showed significant reduction in total oligosaccharides of the raffinose family compared to wild-type seeds. We also found intriguing variations in sucrose, protein and fat content, suggesting the existence of unknown regulatory biases in soybean RFO biosynthesis that merits further investigation. No difference in plant morphology, or seed germination between mutant and wild-type plants was observed under our growth conditions. This study provides new data to further our understanding of the mechanisms of RFO biosynthesis, with the potential to provide the means to significantly improve the nutritional quality of soybean.

## Data Availability Statement

The raw data supporting the conclusions of this article will be made available by the authors, without undue reservation.

## Author Contributions

HC and PD conceived and supervised the study. HC, PD, NP, and HL designed the study. HL, TL, NN, and DT conducted the experiments. CN provided initial training and assisted vector construction. HR provided seed component analysis. TB and NL assisted the experiments. HL and NN analyzed the data. HL, NN, and PD wrote the manuscript. HC, GS, MS, and HR revised and proofread the manuscript. All authors contributed to the article and approved the submitted version.

## Conflict of Interest

The authors declare that the research was conducted in the absence of any commercial or financial relationships that could be construed as a potential conflict of interest.

## Footnotes

1 soykb.org

## Supplementary Material

Supplementary Figure 1 The galactinol and RFO biosynthesis pathway in plant (adapted from Sengupta et al., 2015).

Supplementary Figure 2 Phylogenetic relationships among GOLS proteins in *Arabidopsis, soybean, tomato and maize*. Phylogenetic tree was constructed using MEGA X by the N.J method analysis (1,000 replicates).

Supplementary Figure 3 Transcriptomics analysis for GOLSs in soybean. Data were obtained from soykb.org.

Supplementary Figure 4 T-DNA-based CRISPR/Cas9 vector for soybean transformation, with pFGC5941 backbone, *bar* gene as selection marker, Cas9 (plant-codon optimized Cas9 containing potato IV2 intron) driven by *35S promoter* and two sgRNAs driven by *Arabidopsis U6* promoter *(AtU6-26).* MAS, Manopine promoter; MAS-ter, *Manopine Synthase* terminator; Nos, *Nopaline synthase* promoter; LB/RB – Left and Right Border.

Supplementary Figure 5 Induced mutation analysis of soybean hairy roots using denaturation and renaturation PCR – polyacrylamide gel electrophoresis. Twelve hairy roots generated by *Agrobacterium rhizogenes* transformation method were analyzed for *GmGOLS1A* **(A)** and *GmGOLS1B* **(B)** the target sites. (M) 100 bp DNA ladder; WT, wild-type DT26 sample; lanes 1–12, mixed PCR amplicons of hairy roots lines and WT after denaturation and renaturation.

Supplementary Figure 6 Induced mutations in *GmGOLS1A* **(A)** and *GmGOLS1B* **(B)** in DT1.1 T2 plants. Δ indicates the mutational change: 0 for no change, −for deletion, +for insertion. The genotypes of T2 mutant lines were classified as wt for wild type genotype, biallelic for two different mutant alleles, hetero for heterozygotic with one wild type allele and one mutant allele, homo for homozygotes with two identical mutant alleles.

Supplementary Figure 7 Carbohydrate composition in soybean seeds as proportions of total soluble carbohydrates mass measured, with the major carbohydrates stachyose and sucrose. Wild-type seeds are of DT26 cultivar; DT1.1-7-2, DT1.1-14-3 are seeds from respectively, named T2 gmgols1A single mutant; DT1.1-5-4, DT1.1-7-1 are seeds from T2 gmgols1A gmgols1B double mutants. Statistical analysis was done using one-way ANOVA followed by a *post hoc* Tukey’s multiple range test. Measurements that were not significantly different from one another (*p* > 0.05) share the same letter label (a,b,c). Mean values ± SD for *n* = 4 are shown.

Supplementary Figure 8 Composition of wild-type and mutant soybean seeds excluding soluble carbohydrates. Wild-type seeds are of DT26 cultivar; DT1.1-7-2, DT1.1-14-3 are seeds from respectively, named T2 *gmgols1A* single mutant; DT1.1-5-4, DT1.1-7-1 are seeds from T2 *gmgols1A gmgols1B* double mutants.

Supplementary Figure 9 Morphology of targeted mutant soybean plants in green house; **(A)** 40-day-old plants, **(B)** leaf of 40-day-old plants.

Supplementary Figure 10 Transgene-free analysis of mutant lines of D1.1. Leaves are painted with glufosinate (200 mg/L).

Supplementary Figure 11 Sequencing of potential off-target sites and flanking regions from bulks of genomic DNA of T2 plants (*n* = 6) from respectively, named T1 parents.
