# Supplementary figures and images for: CRISPR/Cas9-Mediated Knockout of Galactinol Synthase-Encoding Genes Reduces Raffinose Family Oligosaccharide Levels in Soybean Seeds
Source: Front Plant Sci. 2020 Dec 17;11:612942. doi: 10.3389/fpls.2020.612942 (PMC7773711; doi:10.3389/fpls.2020.612942)

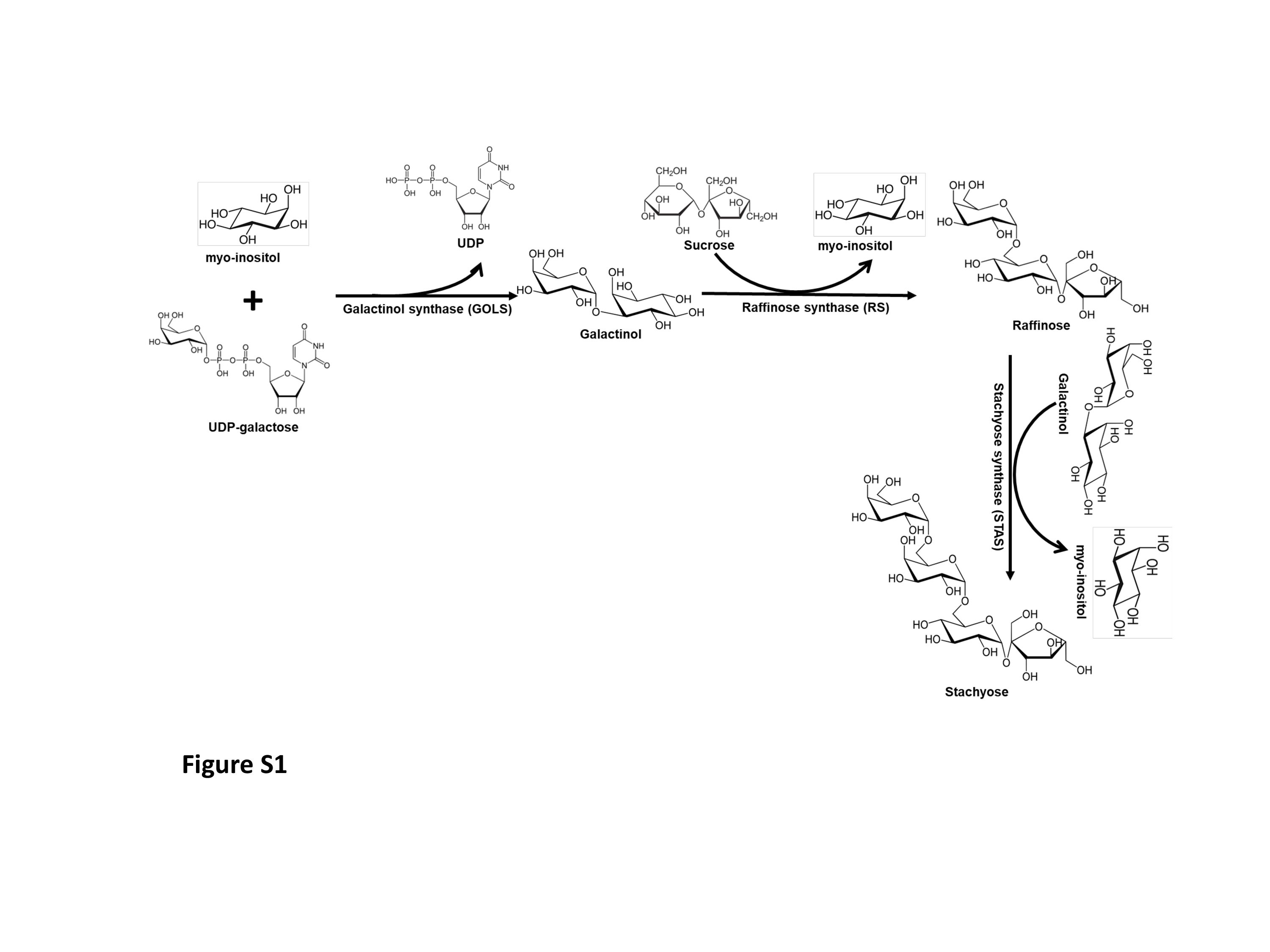

Supplement: Supplementary Figure 1 — The galactinol and RFO biosynthesis pathway in plant (adapted from Sengupta et al., 2015). [file Image_1.JPEG]

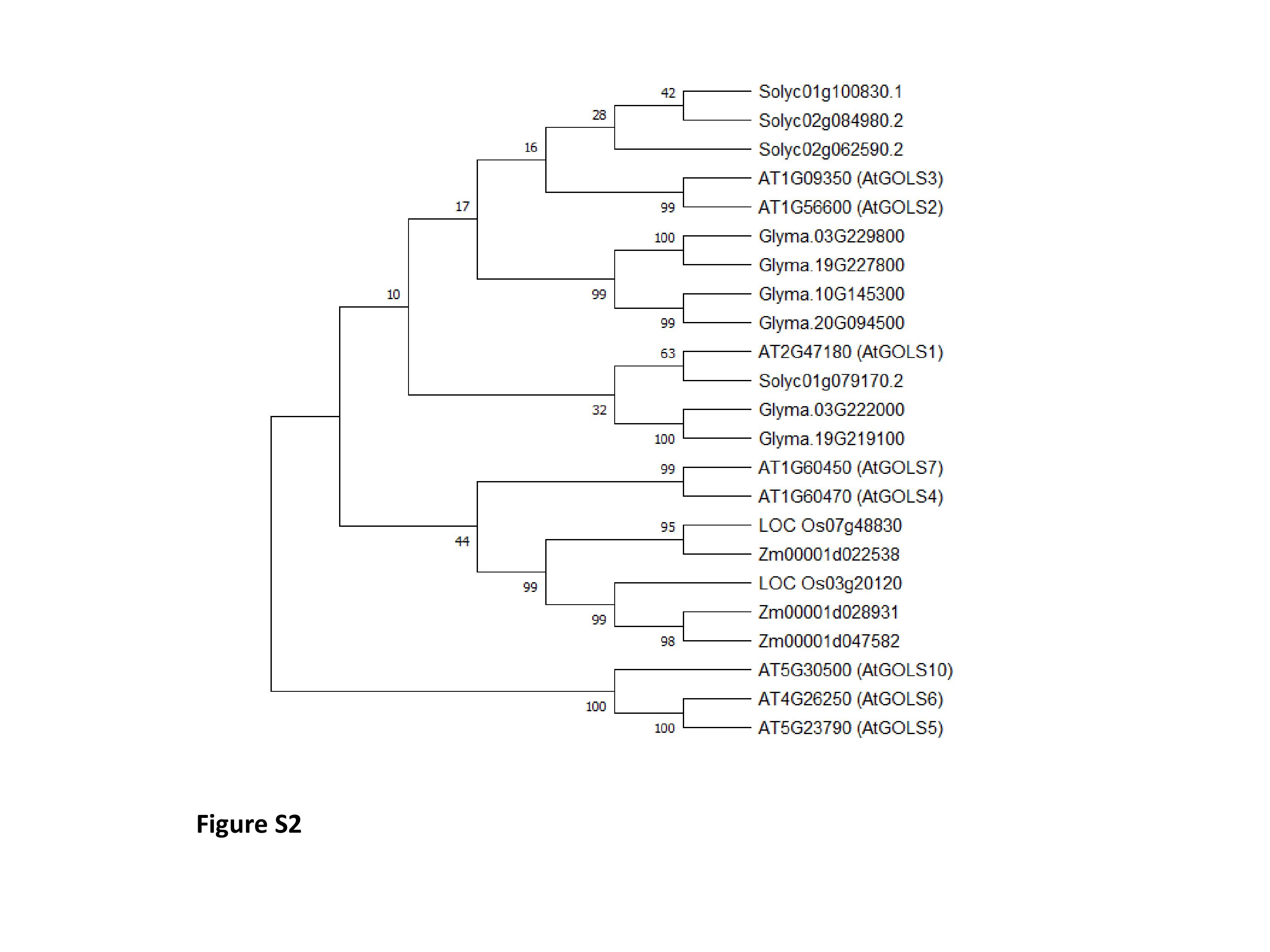

Supplement: Supplementary Figure 2 — Phylogenetic relationships among GOLS proteins in Arabidopsis, soybean, tomato and maize. Phylogenetic tree was constructed using MEGA X by the N.J method analysis (1,000 replicates). [file Image_2.JPEG]

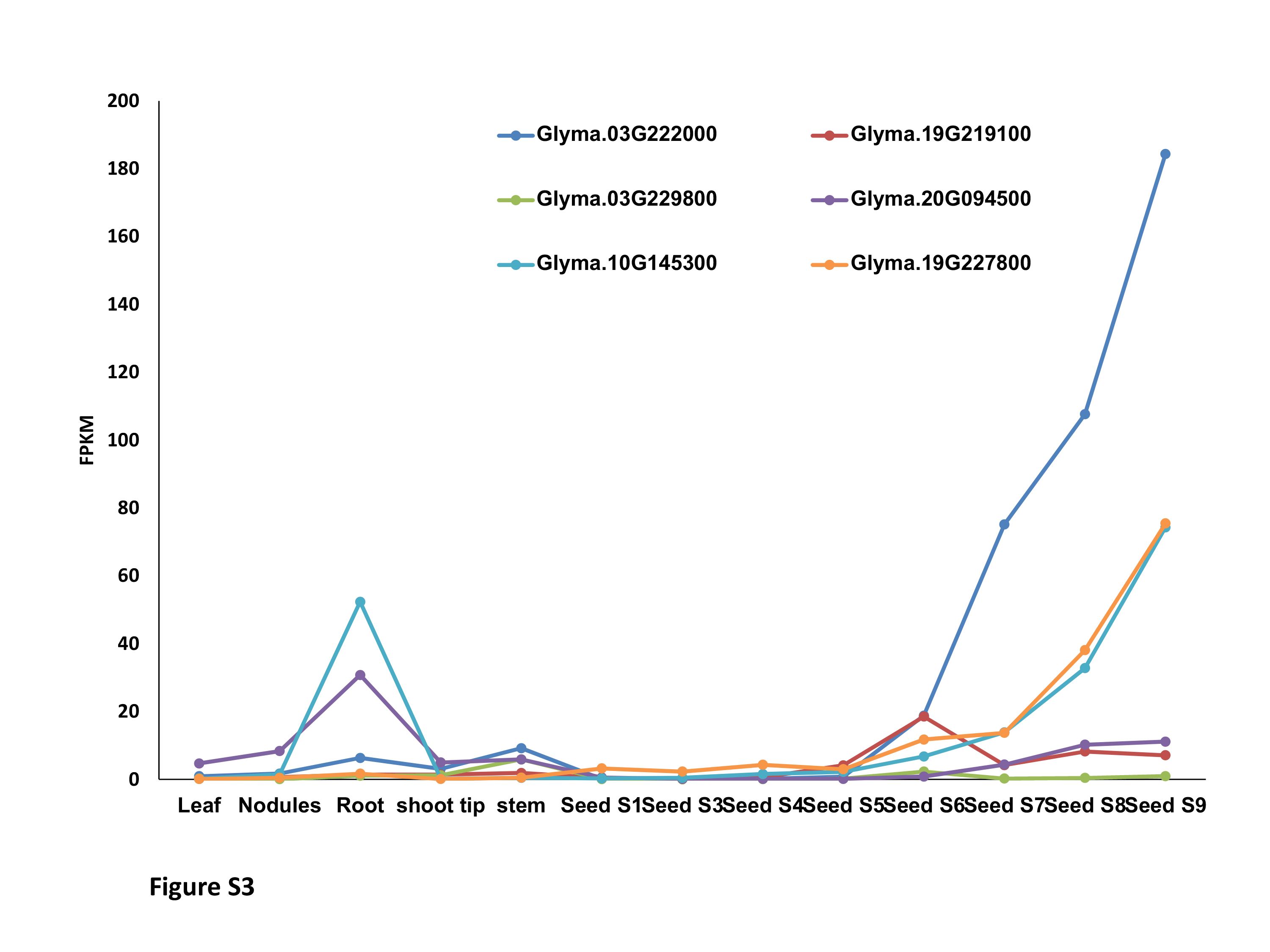

Supplement: Supplementary Figure 3 — Transcriptomics analysis for GOLSs in soybean. Data were obtained from soykb.org. [file Image_3.JPEG]

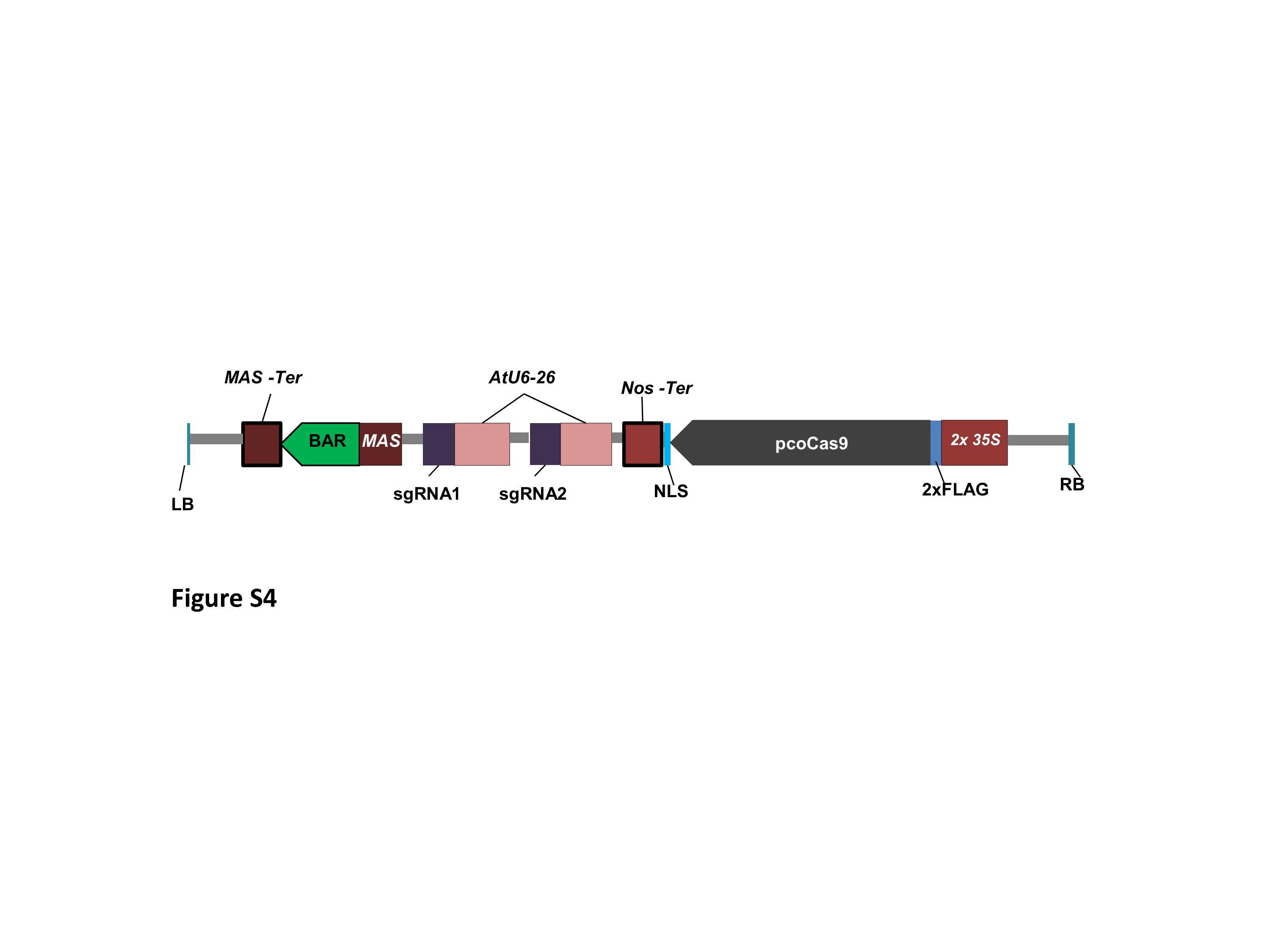

Supplement: Supplementary Figure 4 — T-DNA-based CRISPR/Cas9 vector for soybean transformation, with pFGC5941 backbone, bar gene as selection marker, Cas9 (plant-codon optimized Cas9 containing potato IV2 intron) driven by 35S promoter and two sgRNAs driven by Arabidopsis U6 promoter (AtU6-26). MAS, Manopine promoter; MAS-ter, Manopine Synthase terminator; Nos, Nopaline synthase promoter; LB/RB – Left and Right Border. [file Image_4.JPEG]

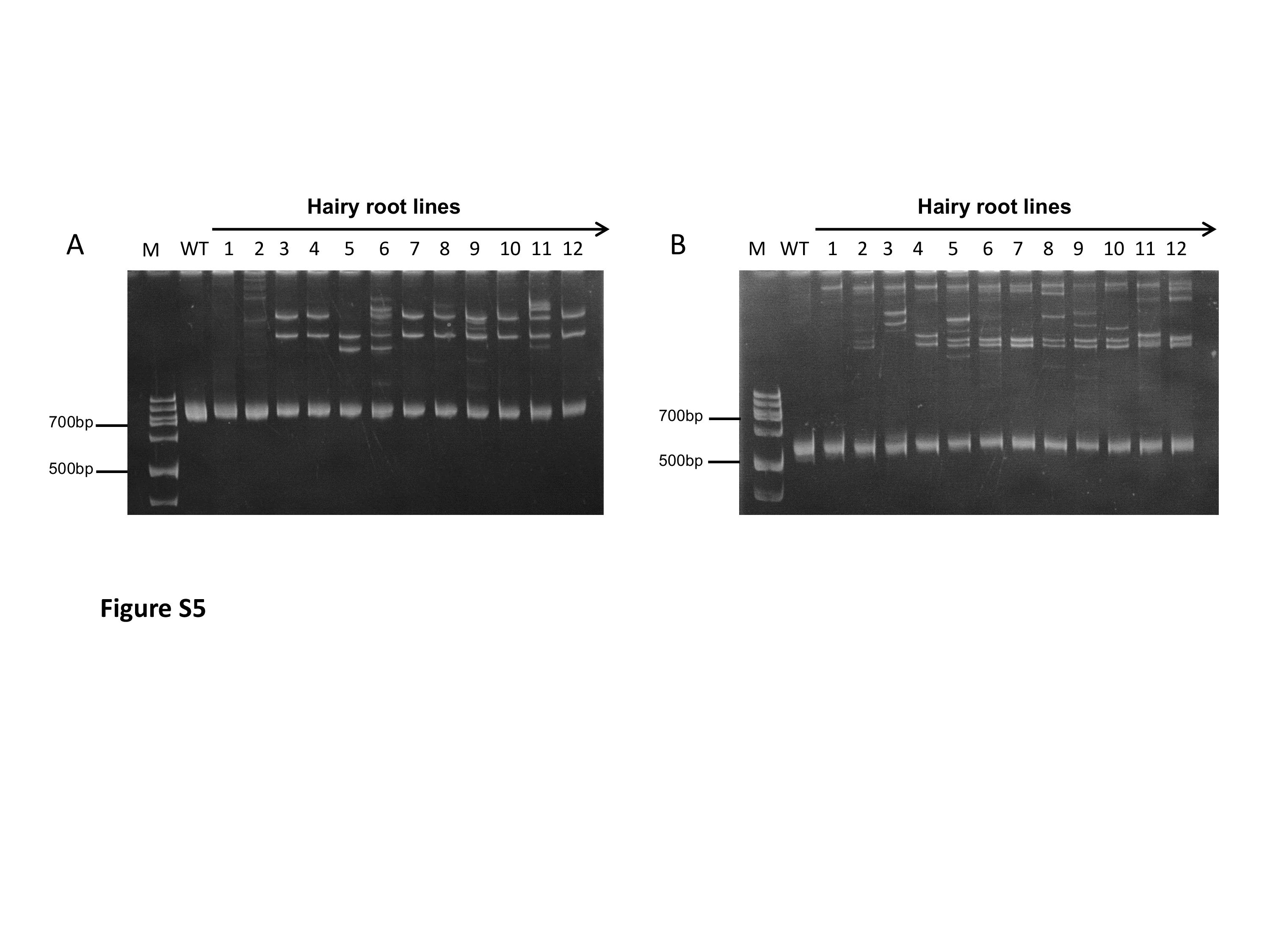

Supplement: Supplementary Figure 5 — Induced mutation analysis of soybean hairy roots using denaturation and renaturation PCR – polyacrylamide gel electrophoresis. Twelve hairy roots generated by Agrobacterium rhizogenes transformation method were analyzed for GmGOLS1A (A) and GmGOLS1B (B) the target sites. (M) 100 bp DNA ladder; WT, wild-type DT26 sample; lanes 1–12, mixed PCR amplicons of hairy roots lines and WT after denaturation and renaturation. [file Image_5.JPEG]

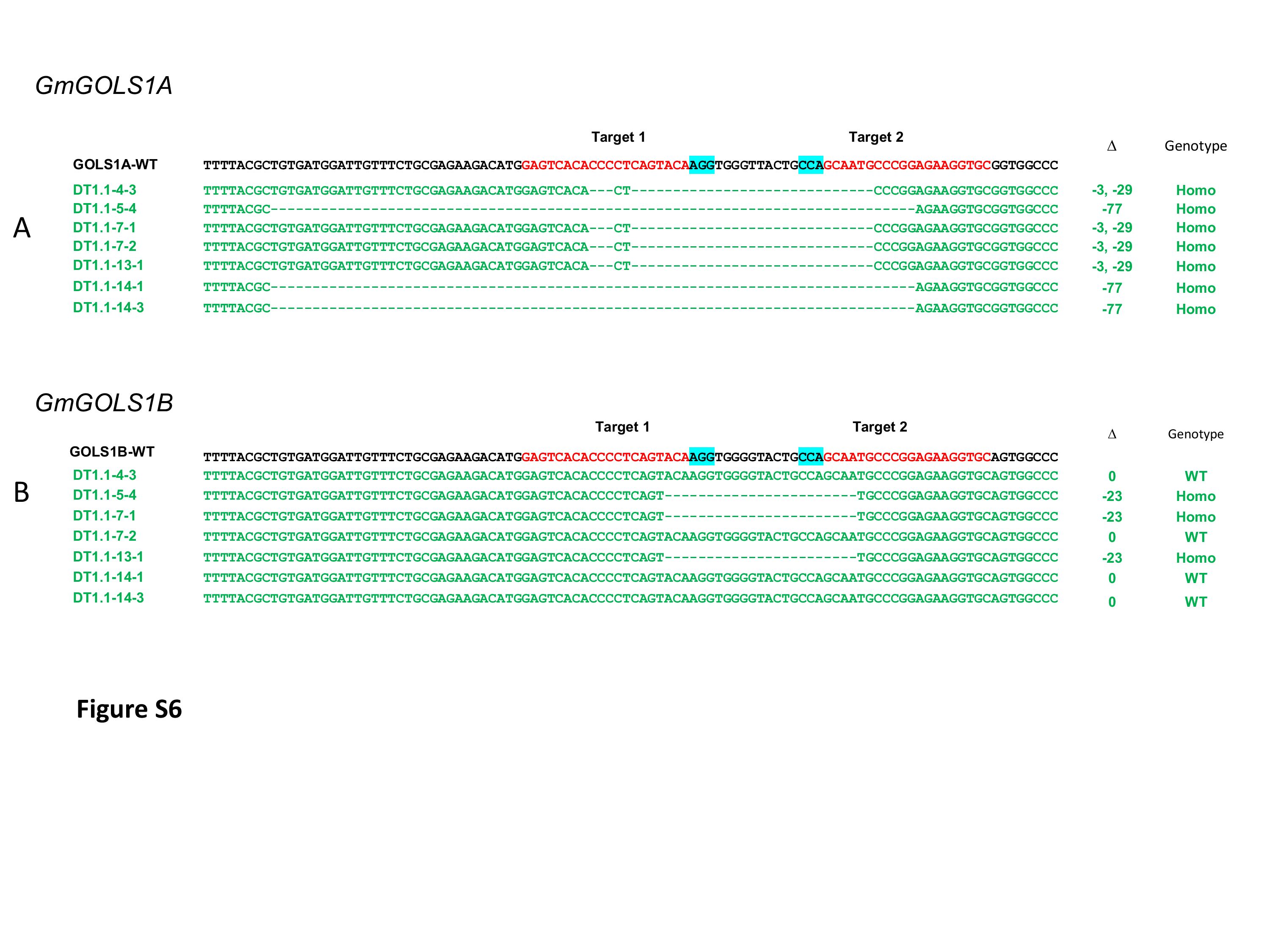

Supplement: Supplementary Figure 6 — Induced mutations in GmGOLS1A (A) and GmGOLS1B (B) in DT1.1 T2 plants. Δ indicates the mutational change: 0 for no change, −for deletion, +for insertion. The genotypes of T2 mutant lines were classified as wt for wild type genotype, biallelic for two different mutant alleles, hetero for heterozygotic with one wild type allele and one mutant allele, homo for homozygotes with two identical mutant alleles. [file Image_6.JPEG]

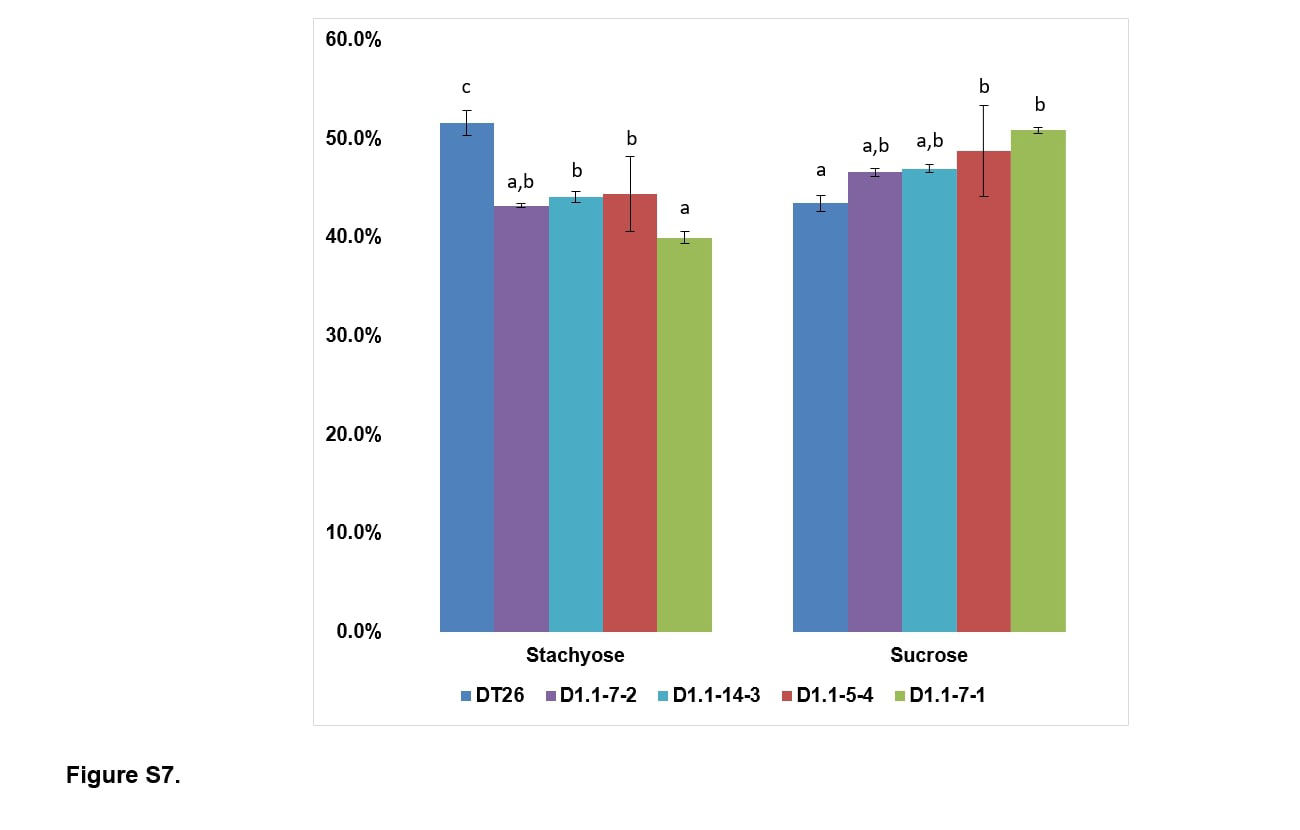

Supplement: Supplementary Figure 7 — Carbohydrate composition in soybean seeds as proportions of total soluble carbohydrates mass measured, with the major carbohydrates stachyose and sucrose. Wild-type seeds are of DT26 cultivar; DT1.1-7-2, DT1.1-14-3 are seeds from respectively, named T2 gmgols1A single mutant; DT1.1-5-4, DT1.1-7-1 are seeds from T2 gmgols1A gmgols1B double mutants. Statistical analysis was done using one-way ANOVA followed by a post hoc Tukey’s multiple range test. Measurements that were not significantly different from one another (p > 0.05) share the same letter label (a,b,c). Mean values ± SD for n = 4 are shown. [file Image_7.jpg]

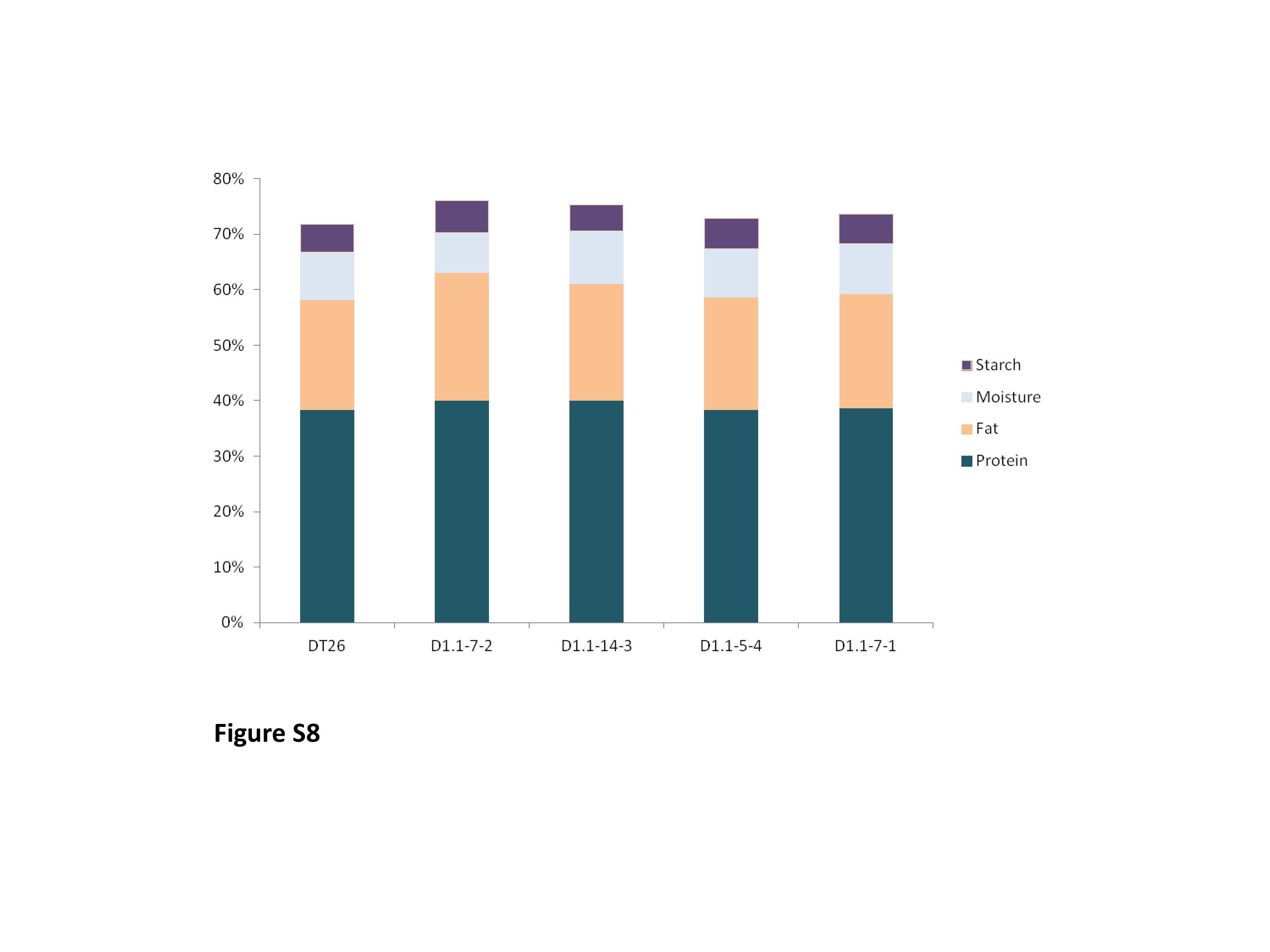

Supplement: Supplementary Figure 8 — Composition of wild-type and mutant soybean seeds excluding soluble carbohydrates. Wild-type seeds are of DT26 cultivar; DT1.1-7-2, DT1.1-14-3 are seeds from respectively, named T2 gmgols1A single mutant; DT1.1-5-4, DT1.1-7-1 are seeds from T2 gmgols1A gmgols1B double mutants. [file Image_8.JPEG]

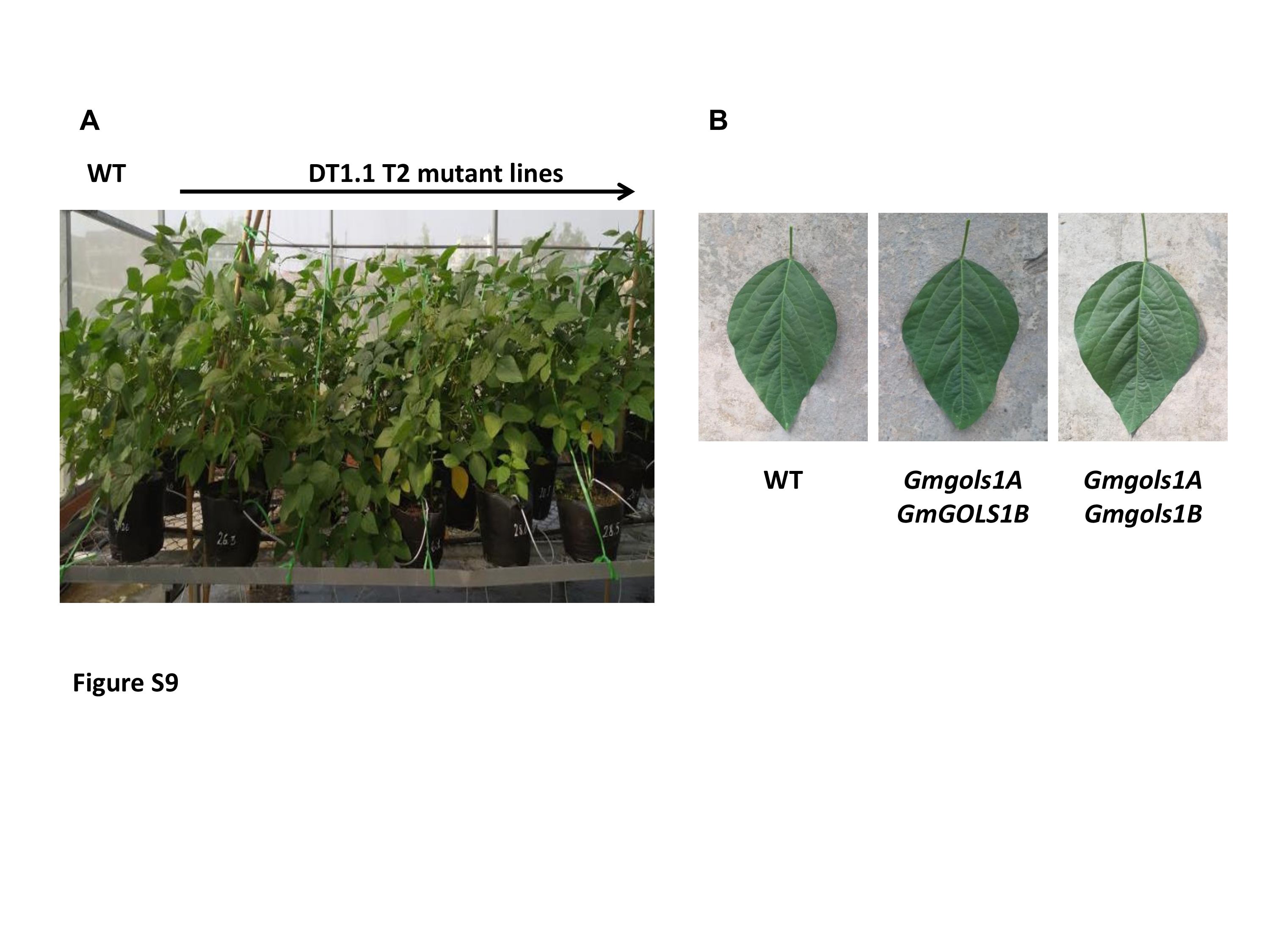

Supplement: Supplementary Figure 9 — Morphology of targeted mutant soybean plants in green house; (A) 40-day-old plants, (B) leaf of 40-day-old plants. [file Image_9.JPEG]

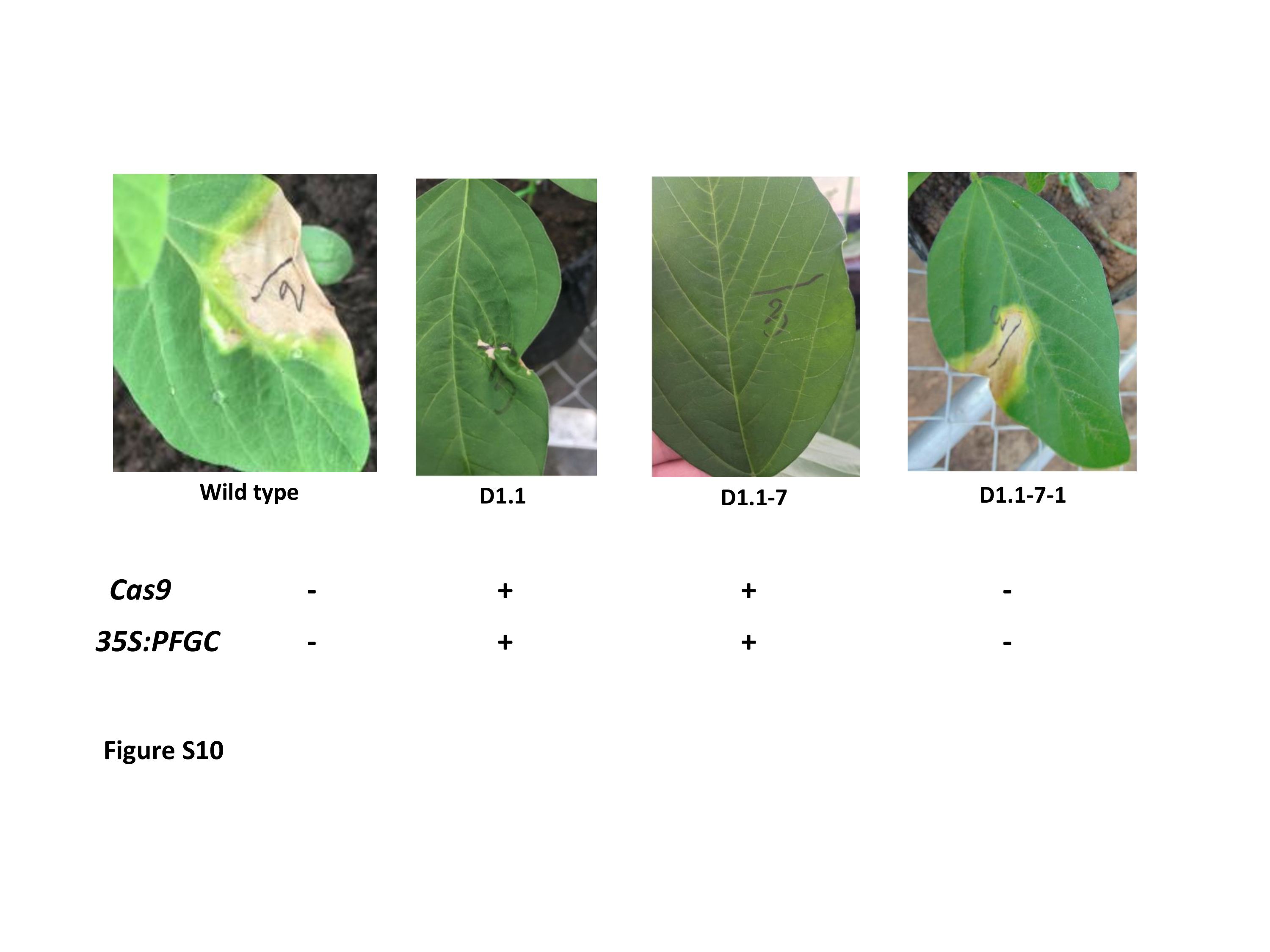

Supplement: Supplementary Figure 10 — Transgene-free analysis of mutant lines of D1.1. Leaves are painted with glufosinate (200 mg/L). [file Image_10.JPEG]

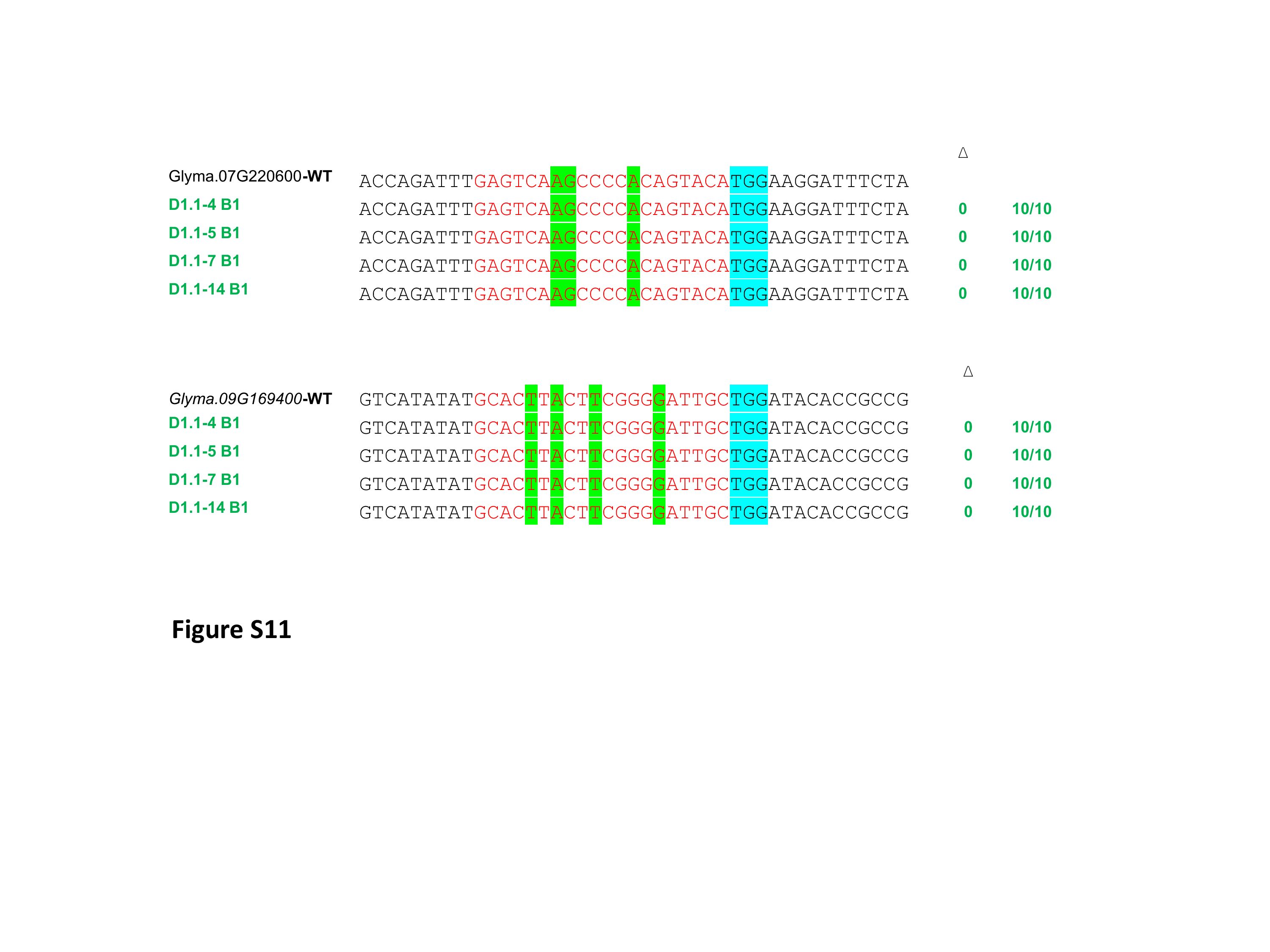

Supplement: Supplementary Figure 11 — Sequencing of potential off-target sites and flanking regions from bulks of genomic DNA of T2 plants (n = 6) from respectively, named T1 parents. [file Image_11.JPEG]
