# Supplementary material for: CRISPR/Cas9-Mediated Knockout of Galactinol Synthase-Encoding Genes Reduces Raffinose Family Oligosaccharide Levels in Soybean Seeds
Source: Front Plant Sci. 2020 Dec 17;11:612942. doi: 10.3389/fpls.2020.612942 (PMC7773711; doi:10.3389/fpls.2020.612942)
Supplement: Supplementary file 12 [file Table_1.DOCX]

The Supplementary Tables

**Table S1.** Sequences of oligonucleotides and primer sets

| **Oligonucleotide** | **5’-3’ sequence** | **Description** |
| --- | --- | --- |
| **sgRNA1** | GAGTCACACCCCTCAGTACA | Annealed to create gRNA for target 1. |
| **sgRNA1c** | TGTACTGAGGGGTGTGACTC |  |
| **sgRNA2** | GCACCTTCTCCGGGCATTGC | Annealed to create gRNA for target 2. |
| **sgRNA2c** | GCAATGCCCGGAGAAGGTGC |  |
| **pcoCas9 F** | TGAGGAGACCATCACCCCTT | Primer pair specific to *pcoCas9* gene. |
| **pcoCas9 R** | AAGTCTACCCCATCCGGTGT |  |
| **35S:pFGC F** | TGTGCGTCATCCCTTACGTC | Primer pair specific to a transgene region spanning pFGC and 35SPPDK promoter. |
| **35S:pFGC R** | GAAGGCGGGAAACGACAATC |  |
| **G03 F** | TGACGGAAATGGCCATGCTCCTG | Primer pair flanking the target region of *GmGOLS1A* |
| **G03 R** | CCCCGTATATCTCCATGGCTTGG |  |
| **G19 F** | TCTTGATTGAGTAAGGTGTGAG | Primer pair flanking the target region of *GmGOLS1B* |
| **G19 R** | GCGCCAGAGCATGGCAAGGAC |  |
| **G07 F** | ATGTACAAACCAGACTGCTGTT | Primer pair flanking potential off-target site on chromosome 07 |
| **G07 R** | GTACATGGCAGGTGACAACCT |  |
| **G09 F** | GCTTGGATCTGGCACTCAGTT | Primer pair flanking potential off-target site on chromosome 09 |
| **G09 R** | GCTTGAAATTCAGGCCTTGCTA |  |

**Table S2:** Composition of wild-type and mutant soybean seeds excluding soluble carbohydrates.

|  | **Protein (%)** | **Fat (%)** | **Starch (%)** |
| --- | --- | --- | --- |
| **WT (DT26)** | 38.30 ± 0.49 a | 19.83 ± 1.28 a | 5.090 ± 0.84 a |
| **D1.1-7-2** | 40.06 ± 1.09 b | 22.88 ± 0.52 b | 5.770 ± 1.00 a |
| **D1.1-14-3** | 40.06 ± 0.54 b | 20.92 ± 0.69 a | 4.775 ± 0.38 a |
| **D1.1-5-4** | 38.28 ± 0.49 a | 20.37 ± 1.45 a | 5.509 ± 1.29 a |
| **D1.1-7-1** | 38.68 ± 0.53 a | 20.52 ± 0.79 a | 5.219 ± 1.19 a |

***Note:*** *Wild-type seeds are of DT26 cultivar; D1.1-7-2, D1.1-14-3 are seeds from respectively named T2 gmgols1A single mutant; D1.1-5-4, D1.1-7-1 are seeds from T2 gmgols1A gmgols1B double mutants. Statistical analysis was done using one-way ANOVA followed by a post-hoc Tukey’s multiple range test. Different letters denote significant differences at p <0.05.*

**Table S3.** Screening result of transgene presence in edited plants. Plants are color-coded according to their T0 events.

| **Generation** | **Lines** | | **Herbicide leaf painting** | | **Cas9** | | **35S:pFGC** | |  |
| --- | --- | --- | --- | --- | --- | --- | --- | --- | --- |
| **T0** | **DT1.1** | | **R** | | **+** | | **+** | |  |
|  | **M3.1** | | **R** | | **+** | | **+** | |  |
|  | **M4.1** | | **R** | | **+** | | **+** | |  |
| **T1** | **DT1.1-1** | | **R** | | **+** | | **+** | |  |
|  | **DT1.1-2** | | **R** | | **+** | | **+** | |  |
|  | **DT1.1-3** | | **R** | | **+** | | **+** | |  |
|  | **DT1.1-4** | | **R** | | **+** | | **+** | |  |
|  | **DT1.1-5** | | **R** | | **+** | | **+** | |  |
|  | **DT1.1-6** | | **R** | | **+** | | **+** | |  |
|  | **DT1.1-7** | | **R** | | **+** | | **+** | |  |
|  | **DT1.1-8** | | **R** | | **+** | | **+** | |  |
|  | **DT1.1-9** | | **R** | | **+** | | **+** | |  |
|  | **DT1.1-10** | | **R** | | **+** | | **+** | |  |
|  | **DT1.1-11** | | **R** | | **+** | | **+** | |  |
|  | **DT1.1-12** | | **R** | | **+** | | **+** | |  |
|  | **DT1.1-13** | | **R** | | **+** | | **+** | |  |
|  | **DT1.1-14** | | **R** | | **+** | | **+** | |  |
|  | **DT1.1-15** | | **R** | | **+** | | **+** | |  |
|  | **DT1.1-16** | | **R** | | **+** | | **+** | |  |
|  | **DT1.1-17** | | **R** | | **+** | | **+** | |  |
|  | **DT1.1-18** | | **R** | | **+** | | **+** | |  |
|  | **DT1.1-19** | | **R** | | **+** | | **+** | |  |
|  | **DT1.1-20** | | **R** | | **+** | | **+** | |  |
|  | **DT1.1-21** | | **R** | | **+** | | **+** | |  |
| **Generation** | | **Lines** | | **Herbicide leaf painting** | | **Cas9** | | **35S:pFGC** | |
| **T1** | | **DT1.1-22** | | **R** | | **+** | | **+** | |
|  |  | **DT1.1-23** | | **R** | | **+** | | **+** | |
|  |  | **DT1.1-24** | | **R** | | **+** | | **+** | |
|  |  | **DT1.1-25** | | **R** | | **+** | | **+** | |
|  |  | **DT1.1-26** | | **R** | | **+** | | **+** | |
|  |  | **DT1.1-27** | | **R** | | **+** | | **+** | |
|  |  | **DT1.1-28** | | **R** | | **+** | | **+** | |
|  |  | **DT1.1-29** | | **R** | | **+** | | **+** | |
|  |  | **M3.1-2** | | **R** | | **+** | | **+** | |
|  |  | **M3.1-5** | | **S** | | **-** | | **-** | |
|  |  | **M3.1-6** | | **R** | | **+** | | **+** | |
|  |  | **M3.1-9** | | **S** | | **-** | | **-** | |
|  |  | **M4.1-1** | | **R** | | **+** | | **+** | |
|  |  | **M4.1-2** | | **S** | | **-** | | **-** | |
|  |  | **M4.1-4** | | **S** | | **-** | | **-** | |
|  |  | **M4.1-5** | | **R** | | **+** | | **+** | |
| **T2** | | **DT1.1-4-3** | | **R** | | **+** | | **+** | |
|  |  | **DT1.1-5-4** | | **R** | | **+** | | **+** | |
|  |  | **DT1.1-7-1** | | **S** | | **-** | | **-** | |
|  |  | **DT1.1-7-2** | | **S** | | **-** | | **-** | |
|  |  | **DT1.1-13-1** | | **R** | | **+** | | **+** | |
|  |  | **DT1.1-13-3** | | **S** | | **-** | | **-** | |
|  |  | **DT1.1-14-1** | | **R** | | **+** | | **+** | |
|  |  | **DT1.1-14-3** | | **R** | | **+** | | **+** | |

*Note: R (Herbicide resistant); S (Herbicide susceptible); + (PCR indicates transgene presence in genome); - (PCR indicates transgene-free genome).*

**Table S4.** Potential off-target mutations in T2 plants.

|  | **Sequence** | **MMs** | **Gene locus** | **Region** | **Identified mutant** |
| --- | --- | --- | --- | --- | --- |
| Target sequence | GAGTCACACCCCTCAGTACA | 0 | Glyma.03G38080  Glyma.19G40680 | Exon |  |
| Potential off-target | GAGTCAAGCCCCACAGTACATGG | 3 | Glyma.07G34570 | UTR | 0 |
| Target sequence | GCACCTTCTCCGGGCATTGC | 0 | Glyma.03G38080  Glyma.19G40680 | Exon |  |
| Potential off-target | GCACTTACTTCGGGGATTGCAGG | 4 | Glyma.09G14090 | Exon | 0 |

*Note: Red letters in sequence indicate mismatch sites; underlined letters in sequence indicate PAM; MMs: Number of mismatches.*
